# Developmental basis of phenotypic integration in two Lake Malawi cichlids

**DOI:** 10.1186/s13227-016-0040-z

**Published:** 2016-01-21

**Authors:** Pierre Le Pabic, W. James Cooper, Thomas F. Schilling

**Affiliations:** Department of Developmental and Cell Biology, 4109 Natural Sciences II, University of California, Irvine, CA 92697-2300 USA; School of Biological Sciences, Washington State University Tri-cities, 2710 Crimson Way, Richland, WA 99354 USA

**Keywords:** Developmental module, Developmental constraint, Phenotypic integration, Skeletal development, Cichlid evolution

## Abstract

**Background:**

Cichlid fishes from the Rift Lakes of East Africa have undergone the most spectacular adaptive radiations in vertebrate history. Eco-morphological adaptations in lakes Victoria, Malawi and Tanganyika have resulted in a vast array of skull shapes and sizes, yet primary axes of morphological variation are conserved in all three radiations, prominently including the size of the preorbital region of the skull. This conserved pattern suggests that development may constrain the trajectories of cichlid head morphological evolution.

**Results:**

Here, we (1) present a comparative analysis of adult head morphology in two sand-dweller cichlids from Lake Malawi with preorbital size differences representative of the main axis of variation among the three lakes and (2) analyze the ontogeny of shape and size differences by focusing on known developmental modules throughout the head. We find that (1) developmental differences between the two species correlate with known developmental modules; (2) differences in embryonic cartilage development result in phenotypically integrated changes among all bones derived from a single cartilage, while differences in dermal bone development tend to influence isolated regions within a bone; and lastly (3) species-specific morphologies appear in the embryo as subtle differences, which become progressively amplified throughout ontogeny. We propose that this amplification takes place at skeletal growth zones, the locations and shapes of which are patterned during embryogenesis.

**Conclusions:**

This study is the most anatomically comprehensive analysis of the developmental differences underlying cichlid skull evolution in the Rift Lakes of East Africa. The scale of our analysis reveals previously unnoticed correlations between developmental modules and patterns of phenotypic integration. We propose that the primary axes of morphological variation among East African cichlid adaptive radiations are constrained by the hierarchical modularity of the teleost head skeleton.

## Background

Dawkins (1988) introduced the term “evolvability” to describe the capacity of a lineage to generate heritable phenotypic variation (see also Alberch 1991) [1, 2]. The manner and degree to which developmental mechanisms determine evolvability is one of the major questions of the extended evolutionary synthesis [3–5]. The evolution of the head has been described as the key innovation that allowed the further diversification and evolutionary success of the vertebrate lineage [6]. However, our understanding of the developmental factors that have enabled the massive morphological and functional diversification of the head remains limited. Despite considerable progress in identifying cranial developmental modules and their underlying gene regulatory networks [7–13], little is known about how these modular units structure evolvability.

Some of the best-characterized cranial developmental modules are the pharyngeal arches, an array of serially homologous, bilateral structures that segment the head ventrally [14]. Their derivatives form the entire facial skeleton, which in ray-finned fishes include the ethmoid, upper and lower jaws, suspensorium, opercular series and gill arches. The modular nature of these arches along the antero-posterior axis has been demonstrated by the segment-specific expression and functions of *Hox* genes in patterning arch identity [15–17]. A second set of genes including, but not limited to, *jag1b*, *nkx3.2* and *hand2* subdivides individual pharyngeal arches into dorsal, intermediate and ventral domains, respectively, that give rise to correspondingly located skeletal elements with specific shapes and sizes [18]. The overlap of these two orthogonal patterning systems defines semiautonomous developmental modules through which individual skeletal elements could evolve independently from each other. Changes affecting higher-level modules may affect several adjacent skeletal elements, resulting in phenotypically integrated changes in shape and size, while, in contrast, changes in lower level modules may affect individual bones. In addition, skeletal modularity depends to some extent on the mode of ossification, e.g., individual cartilages may subdivide into multiple distinct bones, while dermal bones often result from the fusion of independent ossification centers [19]. The hierarchical modularity of the vertebrate head may provide useful clues to understanding how patterns of head evolution have been shaped by the developmental constraints imposed by this organization.

Rapid diversification in cranial form has repeatedly generated extensive ecological diversity in multiple lineages of cichlid fishes, which makes them ideal for exploring the developmental properties associated with evolvability [20–24]. Cichlids from the Great East African Rift Lakes (Victoria, Malawi and Tanganyika) have produced some of the most spectacular adaptive radiations in vertebrate history. Their cranial skeletons have evolved extremely quickly, and this has facilitated the invasion of an extraordinary diversity of trophic niches [20–24]. Young et al. [24] and Cooper et al. [22] showed that the primary axes of evolutionary divergence in all three lakes were strongly associated with the size of the preorbital region, suggesting that developmental constraints may have played an important role in shaping their skull evolution.

The adaptive radiation of the Lake Malawi cichlids is currently at a particularly interesting and useful stage for evolutionary developmental biologists. Although it is still very young, a tremendous range of skull shapes has already arisen. The evolution of skull diversity has outpaced the evolution of absolute barriers to interbreeding, such that many species with extremely different cranial morphologies will interbreed in captivity. This dramatically facilitates using both developmental and genetic mapping approaches to study cichlid head evolvability [25–29].

There are two broad categories of environments in Lake Malawi that are accessible to cichlids, and early in their evolutionary history, this lineage split into two sister clades, each of which then diversified further within one of these realms. The names of these lineages reflect their primary habitat use: the *mbuna*, or rock-dwellers, and the sand-dwellers [30]. The major evolutionary axis for both of these clades mirrors the pattern of morphological divergence seen in all three lakes (i.e., diversification in preorbital size and jaw length; [22, 24]). Genetic and developmental investigations of Lake Malawi cichlid skull evolution have primarily focused on isolated modules, such as the jaw skeleton in *mbuna* species [25, 26, 31–36]. In order to test whether the hierarchical modularity of the cichlid head has constrained its evolution, we conducted the most anatomically complete comparative analysis of cichlid head skeleton development to date. By comparing the cranial development of two sand-dweller species with extreme differences in skull morphology both to each other and to the offspring of a hybrid cross between them, we were able to gain insight into the ontogenetic basis of their divergent morphologies. Since the parent species exhibit strong differences in preorbital size and jaw length, this study has strong relevance to determining the developmental factors that have shaped the cichlid radiations within the Great East African Rift Lakes.

We examined the morphological divergence and craniofacial development of *Copadichromis azureus* (CA), which is an omnivorous cichlid with small jaws [37]; and *Dimidiochromis compressiceps* (DC), also known as the “Malawi eye-biter,” which is a predator of small fishes (mainly juveniles of *Utaka* or other shoaling species) with a large mouth and fast biting jaws [38]. We find that morphological differences in adult head shape originate from a few centers that correlate with known developmental modules. Ontogenetic divergence in skeletal shape and/or size is detected for many elements at embryonic and larval stages. As expected, phenotypically integrated differences in adult bones derived from a common cartilage precursor seem to originate from differences in the development of the precursor cartilage itself. In contrast, differences in adult dermal bone morphology seem more independent from each other, which correlates with their more autonomous development. Our observations also indicate that large differences in adult morphology start as subtle module-specific differences in skeletal shape, which are amplified at subsequent stages through differential growth. This reveals a previously unsuspected role for skeletal growth zones in the morphological divergence of cichlids, which will form the focus of our future developmental and genetic mapping studies of cichlid head evolvability.

## Results and discussion

### Morphological differences between the heads of *D. compressiceps*, *C. azureus* and their hybrid progeny

#### Head size and shape differences

**Fig. 1:**
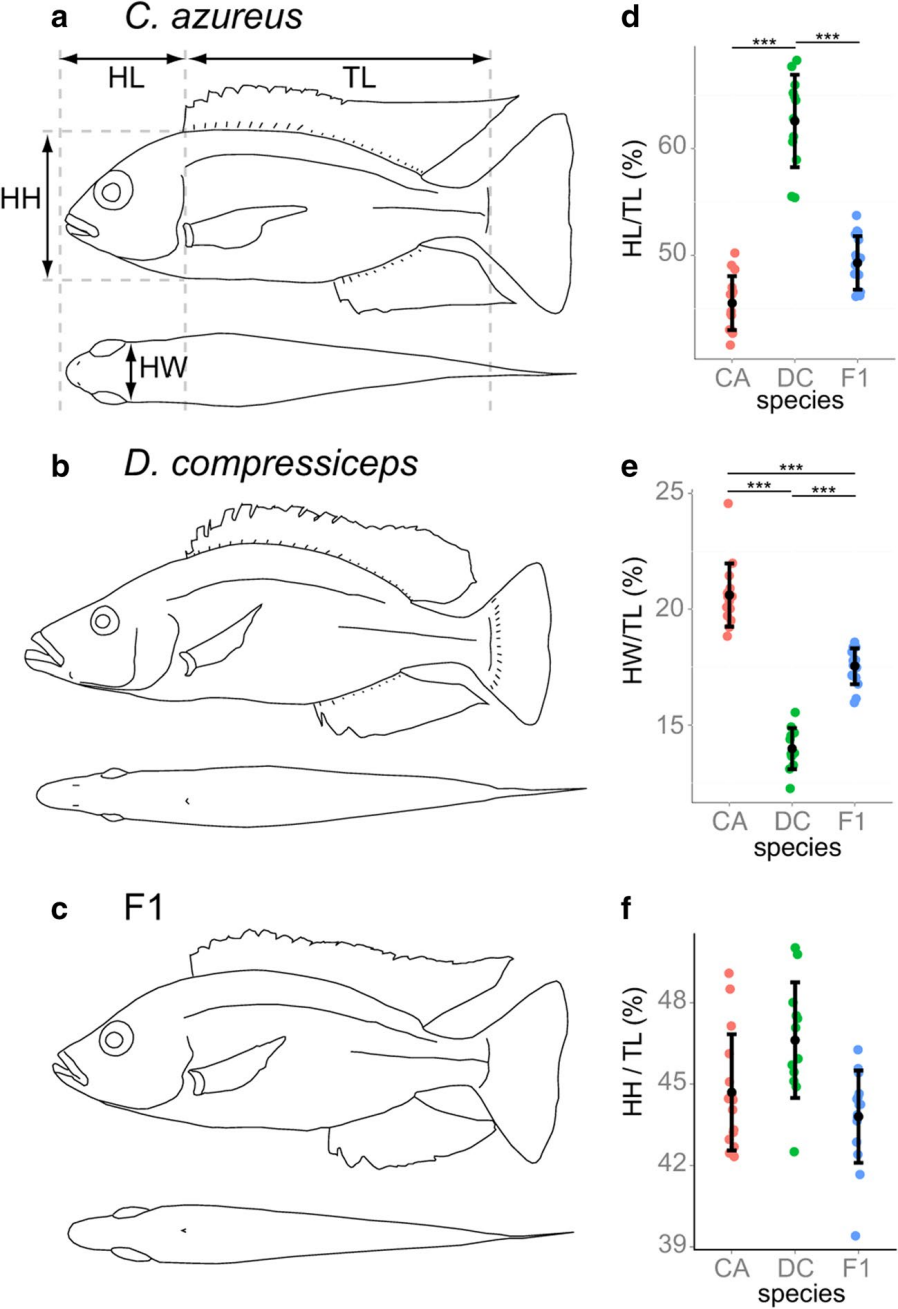
Head length, width and height in *Copadichromis azureus* (CA), *Dimidiochromis compressiceps* (DC) and F1 hybrids. **a**–**c** Camera Lucida drawings of CA, DC and F1 individual. **d** Mean and distribution of head length (HL)/trunk length (TL) for CA (*n* = 15), DC (*n* = 12) and F1 (*n* = 16). **e** Mean and distribution of head width (HW)/TL for CA, DC and F1. **f** Mean and distribution of head height (HH)/TL for CA, DC and F1

Differences in head length (HL), head height (HH) and head width (HW) are expressed as a percentage of trunk length (TL), which is a better normalizing factor than standard length (SL) here as it excludes HL (Fig. 1). Both species differ significantly in HL and HW (Tukey’s test of honest significant difference (HSD) *p* < 0.001; Fig. 1a, b, d, e; Table 1), where the longer (63 % TL), narrower DC head (14 % TL) contrasts with the shorter (45 % TL), wider head of CA (20 % TL). The HL of the F1 hybrids (49 % TL) is closer to that of CA, which suggests dominance of CA alleles for this trait, while intermediate F1 HW values (17 % TL) suggest codominance of parental alleles (Fig. 1c–e). In contrast, HH does not differ significantly between CA (45 % TL), DC (47 % TL) and F1 hybrids (44 % TL; Fig. 1f; Table 1).

**Table 1:** Adult measurements and statistics

	Value	Value as % of CA	Tukey’s HSD *p* value		*p* < 0.001
*Whole body*					
HL/TL (%)					
CA	45.5	100.0	DC–CA	0	***
DC	62.6	137.6	CA–F1	0.0047929	
F1	49.3	108.4	DC–F1	0	***
HW/TL (%)					
CA	20.4	100.0	DC–CA	0	***
DC	14	68.6	CA–F1	0	***
F1	17.5	85.8	DC–F1	0	***
HH/TL (%)					
CA	45	100.0	DC–CA	0.0439263	
DC	46.6	103.6	CA–F1	0.4426099	
F1	43.7	97.1	DC–F1	0.0021476	
*Neurocranium*—*lateral*					
LM 9–16 (cm)					
CA	0.454	100.0	DC–CA	0	***
DC	0.87	191.6	CA–F1	0	***
F1	0.701	154.4	DC–F1	0	***
*Neurocranium*—*dorsal*					
LM 1–4 (cm)					
CA	0.875	100.0	DC–CA	0.00E+00	***
DC	0.59	67.4	CA–F1	0.00E+00	***
F1	0.76	86.9	DC–F1	1.60E−06	***
LM 3–5 (cm)					
CA	1.2	100.0	DC–CA	0	***
DC	1.755	146.2	CA–F1	0	***
F1	1.503	125.3	DC–F1	0	***
*Upper jaw*					
LM 15–18 (cm)					
CA	0.942	100.0	DC–CA	0.0098987	
DC	1.006	106.7	CA–F1	0.0883702	
F1	0.984	104.5	DC–F1	0.5250552	
LM 17–18 (cm)					
CA	0.868	100.0	DC–CA	0.000007	***
DC	0.975	112.4	CA–F1	0.0000001	***
F1	0.99	114.1	DC–F1	0.7184184	
Angle (°)					
CA	82.244	100.0	DC–CA	0	***
DC	71.795	87.3	CA–F1	0.0000004	***
F1	74.241	90.3	DC–F1	0.172744	
*Lower jaw*					
LM 2–3 (cm)					
CA	0.491	100.0	DC–CA	0.00E+00	***
DC	0.489	99.7	CA–F1	2.70E−06	***
F1	0.5	101.8	DC–F1	6.07E−05	***
LM 1–2 (cm)					
CA	0.126	100.0	DC–CA	0	***
DC	0.38	301.8	CA–F1	0.0021672	
F1	0.176	85.8	DC–F1	0	***
LM 4–5 (cm)					
CA	1.026	100.0	DC–CA	0.9954273	
DC	1.307	127.4	CA–F1	0.8679049	
F1	1.175	85.8	DC–F1	0.8364967	
*Suspensorium*					
LM 4–6 (cm)					
CA	1.462	100.0	DC–CA	0	***
DC	1.846	126.3	CA–F1	0	***
F1	1.726	85.8	DC–F1	0.0065184	
LM 4–16 (cm)					
CA	0.993	100.0	DC–CA	0.0059836	
DC	1.068	107.5	CA–F1	0.0635394	
F1	1.042	85.8	DC–F1	0.4975703	
CH length (cm)					
CA	1.168	100.0	DC–CA	0.0004644	***
DC	1.309	112.1	CA–F1	0.5275445	
F1	1.139	85.8	DC–F1	0.0000847	***
*Gill arches*					
CB1 (cm)					
CA	1.014	100.0	DC–CA	0.0000012	***
DC	1.326	130.8	CA–F1	0.1081678	
F1	1.085	85.8	DC–F1	0.0000178	***
Raker spacing (cm)					
CA	0.072	100.0	DC–CA	0.0000007	***
DC	0.117	161.4	CA–F1	0.0963697	
F1	0.082	85.8	DC–F1	0.0000094	***
LTP surface area (cm^2^)					
CA	0.074	100.0	DC–CA	0.0012159	
DC	0.06	82.0	CA–F1	0.0936795	
F1	0.067	85.8	DC–F1	0.0705696	
LTP angle (°)					
CA	64.722	100.0	DC–CA	0.00E+00	***
DC	45.722	70.6	CA–F1	7.00E−07	***
F1	55.153	85.8	DC–F1	9.00E−07	***
LTP tooth spacing (mm)					
CA	0.187	100.0	DC–CA	0.0001263	***
DC	0.253	135.5	CA–F1	0.0007875	***
F1	0.141	85.8	DC–F1	0.0000003	***
UTP surface area (cm^2^)					
CA	0.06	100.0	DC–CA	0.0000159	***
DC	0.038	63.6	CA–F1	0.2056407	
F1	0.055	85.8	DC–F1	0.0002116	***
UTP tooth spacing (mm)					
CA	0.247	100.0	DC–CA	0.1079726	
DC	0.28	113.0	CA–F1	0.0003853	***
F1	0.172	85.8	DC–F1	0.0000155	***

**Fig. 2:**
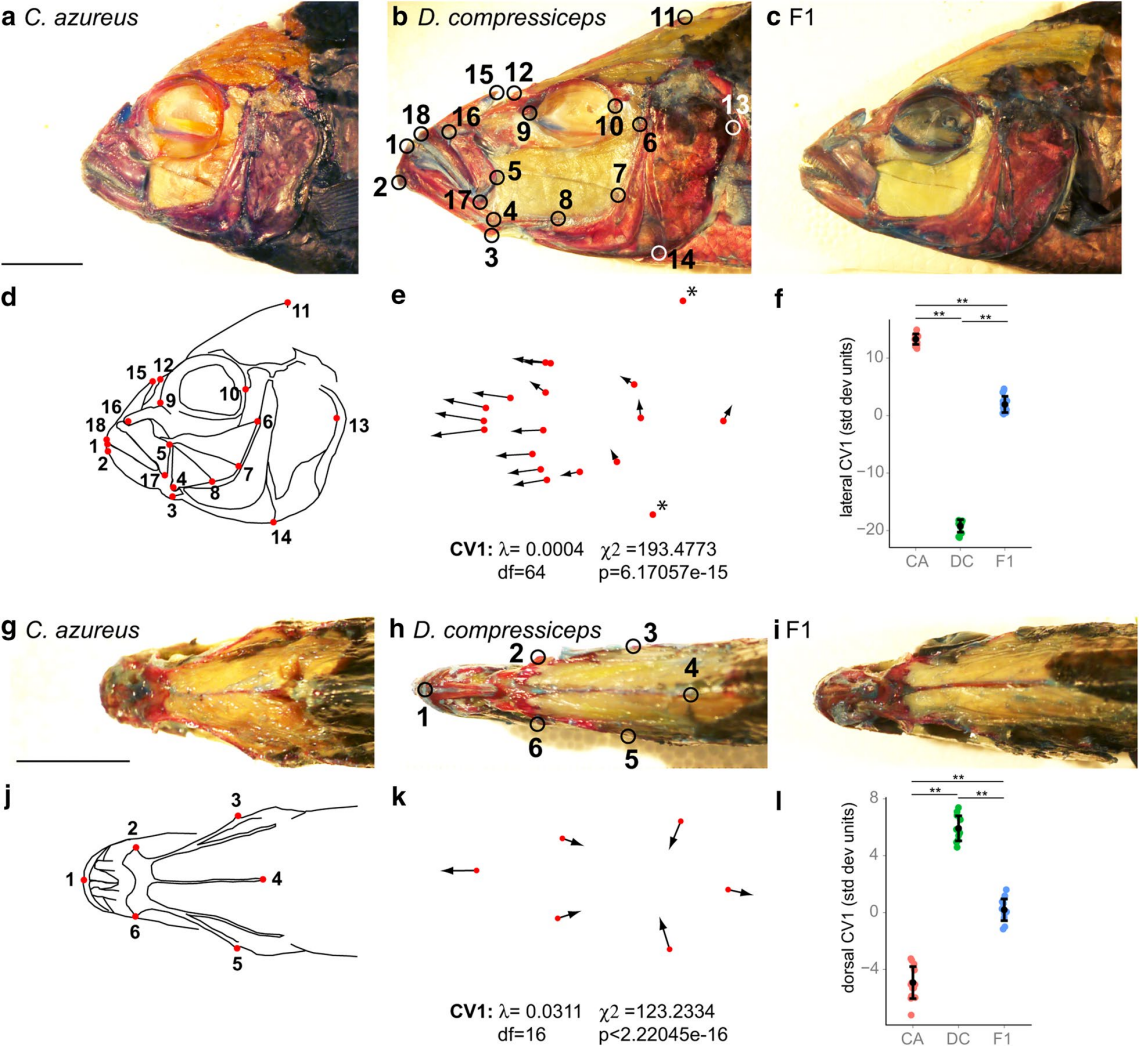
Morphometric analysis of head shape differences between CA, DC and F1 hybrids. **a**–**c** *Lateral views* of the skinned and alizarin red-/alcian blue-stained heads of CA (**a**), DC (**b**) and F1 (**c**) specimens. *Scale bar* 1 cm. **b** Anatomical landmarks examined: *1* anterior tip of the dentary, *2* antero-ventral corner of the dentary, *3* insertion of the interopercular ligament on the articular, *4* articular-quadrate joint (lower jaw joint); *5* postero-dorsal corner of the maxilla, *6* dorsal-most point on the origin of the A1 subdivision of the adductor mandibulae (jaw closing) muscle on the preopercular, *7* dorsal-most point on the origin of the A2 division of the adductor mandibulae on the preopercular, *8* ventral-most point on the origin of the A2 division of the adductor mandibulae on the preopercular, *9* antero-ventral edge of the eye socket, *10* postero-ventral edge of the eye socket, *11* dorsal-most tip of the supraoccipital crest on the neurocranium, *12* joint between the nasal bone and the neurocranium, *13* posterior-most point of the operculum, *14* ventral-most point of overlap between the interoperculum and the suboperculum, *15* posterior tip of the ascending process of the premaxilla, *16* maxillary-palatine joint (upper rotation point of the maxilla), *17* postero-ventral corner of the maxilla, *18* anterior tip of the premaxilla. **d** Anatomical landmarks shown on CA head Camera Lucida drawing. **e** First canonical variate (CV1) deformation vectors on CA landmark configuration. *Asterisks* indicate Bookstein landmarks. **f** Mean and distribution of CA (*n* = 15), DC (*n* = 12) and F1 (*n* = 16) individuals along the CV1 axis, expressed in within-species standard deviation units. **g**–**i** Dorsal views of skinned and alizarin red-/alcian blue-stained heads of CA (**g**), DC (**h**) and F1 (**i**) specimens. *Scale bar* 1 cm. **j** Anatomical landmarks examined shown in **h**: *1* anterior tip of the premaxilla, *2* and *6* lateral-most point in the preorbital region of the frontal, *3* and *5* lateral-most point in the postorbital neurocranium (sphenotic); *4* posterior tip of the supraoccipital crest. **j** Anatomical landmarks shown on CA head Camera Lucida drawing. **k** CV1 deformation vectors on CA landmark configuration. **l** Mean and distribution of CA (*n* = 15), DC (*n* = 12) and F1 (*n* = 16) individuals along the CV1 axis, expressed in within-species standard deviation units. **p < 0.001, Bartlett’s test

In order to determine which aspects of skull anatomy contribute to the observed morphological differences, we quantified shape variation between CA and DC using a morphometric analysis where axes of significant divergence were identified by canonical variate analysis (CVA). Eighteen anatomical landmarks were used on lateral views (Fig. 2b, d) and six landmarks on dorsal views (Fig. 2h, j). Lateral landmarks were largely based on [39] and selected to represent major aspects of the functional morphology of fish feeding. The CVA returned two axes of significant morphological difference between CA and DC for the lateral landmark data (Axis 1: Wilk’s *λ* = 0.0004, *χ*^2^ = 193.4773, *df* = 64, *p* = 6.17057e−15; Axis 2: Wilk’s *λ* = 0.0490, *χ*^2^ = 73.8914, *df* = 31, *p* = 2.31768e−05), which also distinguished each of them from their F1 hybrid progeny, with F1 lateral shape being intermediate between CA and DC (Fig. 2f).

Because of the similar HH between CA, DC and their F1 hybrids (Fig. 1f), landmarks best approximating head height (landmarks 11, 14; Fig. 1d) were set as Bookstein landmarks (asterisks) in order to visualize more precisely the deformation described by CV1 on all other landmarks (Fig. 2e). A clear pattern of anterior deformation is spatially restricted to all landmarks located anterior to the eye in the CA configuration (Fig. 2e, landmarks 1–5, 9, 12, 15–18), indicating a marked trend in anterior expansion of the preorbital region in DC in comparison with CA.

Dorsal head shape differences between CA and DC were also discriminated by CVA, with a single statistically significant axis (Wilk’s *λ* = 0.0311, *χ*^2^ = 123.2334, *df* = 16, *p* < 2.22045e−16). Here, the antero-posterior (A-P) component of CV1 deformation vectors on preorbital landmarks 1, 2 and 6 of the CA configuration is indicative of an anteriorly elongated preorbital region in DC, while the lateral component of the CV1 deformation vectors on landmarks 2, 3, 5 and 6 indicates a narrower head in DC. As with the lateral measures of morphology (Fig. 2f), dorsal F1 shape was intermediate between CA and DC (Fig. 2l).

The results of these analyses confirm that the strong cranial differences in shape between CA and DC are largely restricted to differences in the size of the preorbital region. The skull shapes of CA and DC are therefore widely separated by the same shape axis that describes the primary component of cichlid cranial evolution that arose convergently among both the *mbuna* and sand-dwellers of Lake Malawi and among the cichlid radiations in lakes Victoria and Tanganyika as well.

#### Neurocranium

**Fig. 3:**
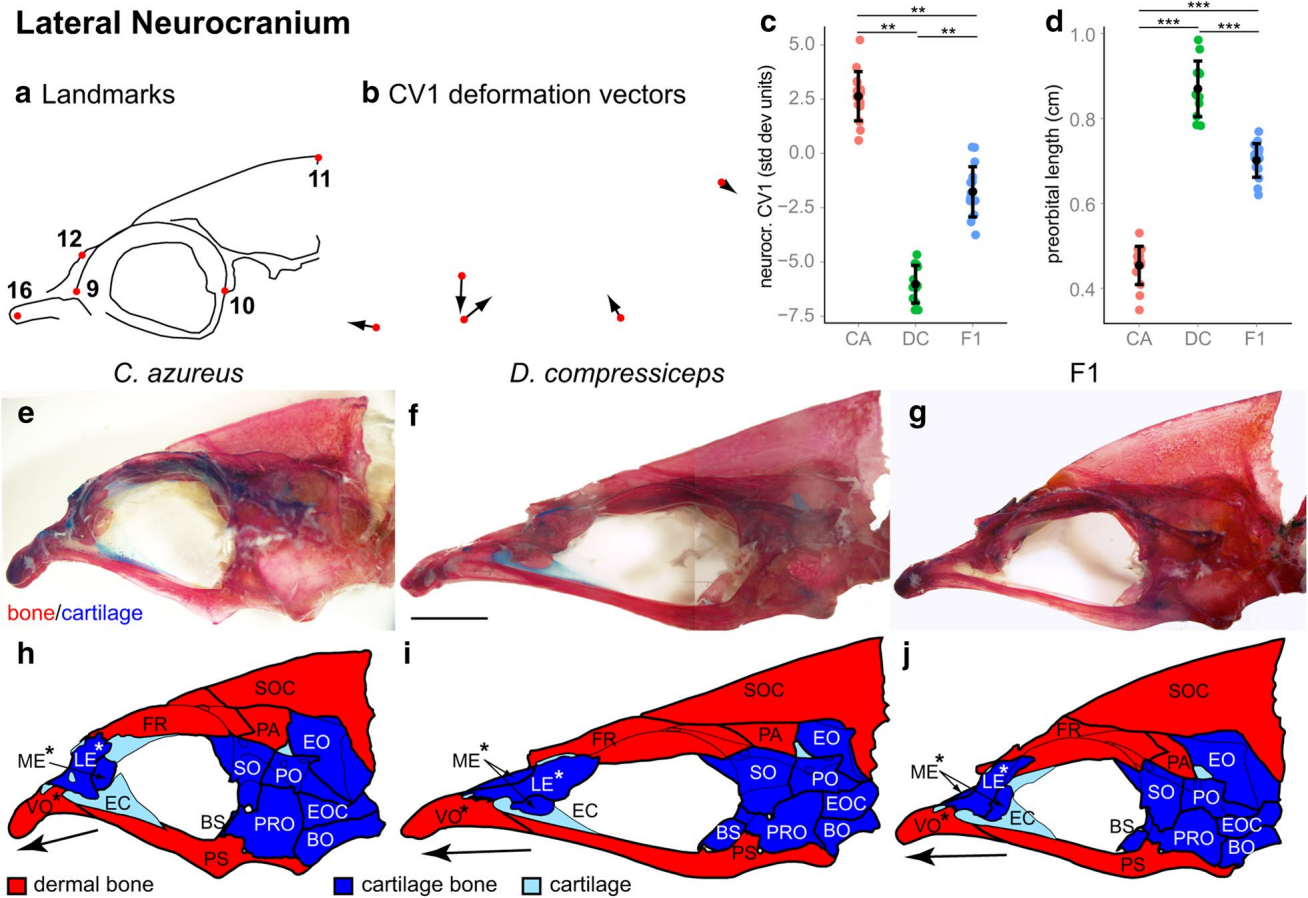
Shape and size differences in the lateral aspect of the neurocranium between CA, DC and F1 hybrids. **a** Camera Lucida drawing of CA neurocranium indicating landmarks. **b** CV1 deformation vectors on CA landmark configuration. **c** Mean and distribution of CA (*n* = 15), DC (*n* = 12) and F1 (*n* = 16) individuals along the neurocranial CV1 axis, expressed in within-species standard deviation units. **p < 0.001, Bartlett’s test. **d** Mean and distribution of CA, DC and F1 preorbital length (cm). **e**–**g** Dissected, alizarin red-/alcian blue-stained neurocrania of CA (**e**), DC (**f**) and F1 (**g**) specimens. *Scale bar* 5 mm. **h**–**j** Individual neurocranial bones labeled on Camera Lucida drawings of CA (**h**), DC (**i**) and F1 (**j**). *Asterisk* (*) indicates preorbital bone. ***p < 0.001, Tukey’s HSD test. Bone nomenclature after [64]. *BO* basioccipital, *BS* basisphenoid, *EC* ethmoid cartilage, *EO* epiotic, *EOC* exoccipital, *FR* frontal, *LE* lateral ethmoid, *ME* mesethmoid, *PA* parietal, *PO* pterotic, *PRO* prootic, *PS* parasphenoid, *SO* sphenotic, *SOC* supraoccipital, *VO* vomer

**Fig. 4:**
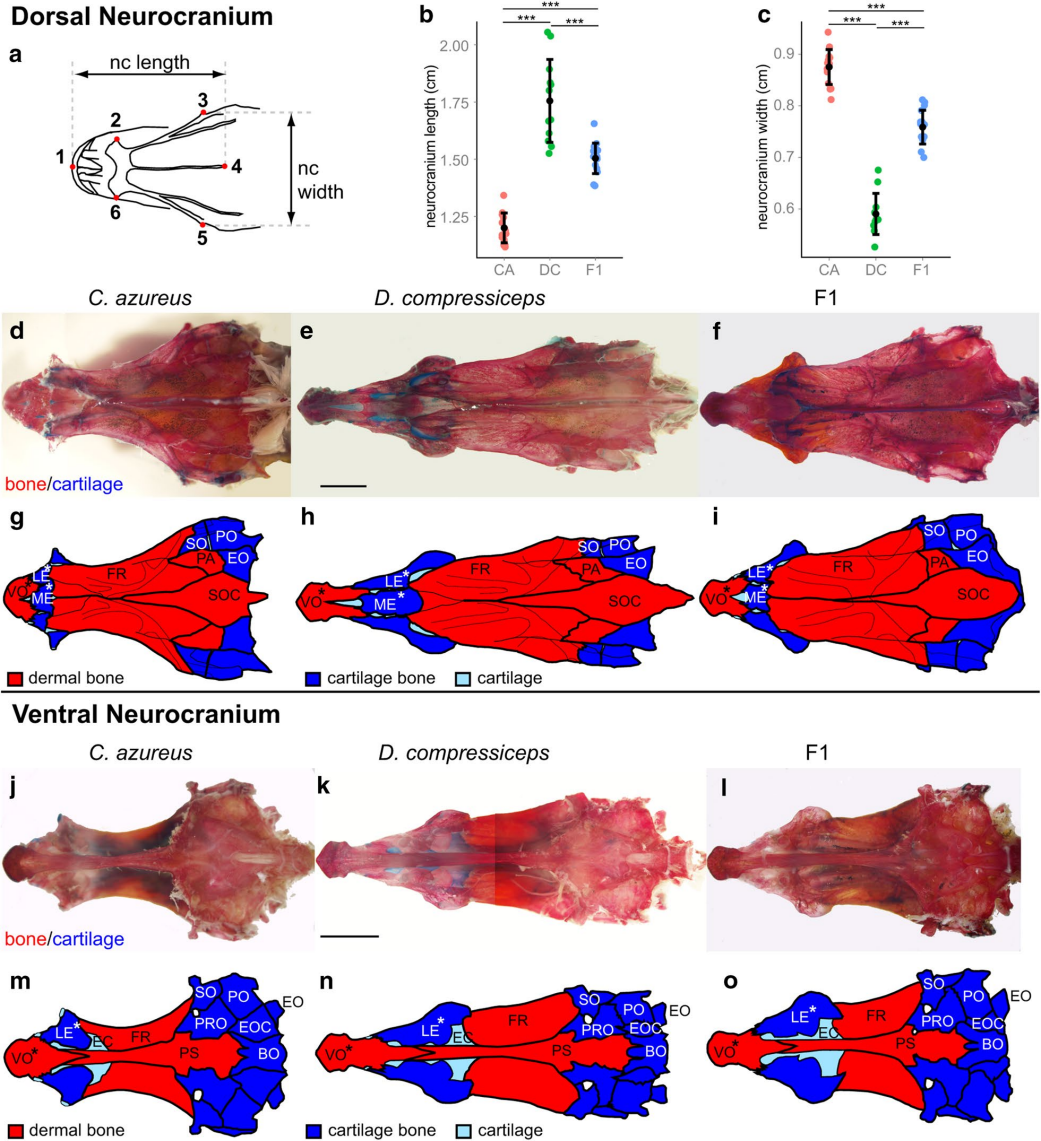
Shape and size differences in the dorsal and ventral aspects of the neurocranium between CA, DC and F1 hybrids. **a** Camera Lucida drawing of CA neurocranium indicating landmarks used for measurements of neurocranial length (**b**) and width (**c**). **b**, **c** Mean and distribution of CA (*n* = 15), DC (*n* = 12) and F1 (*n* = 16) neurocranial length (**b**) and width (**c**). **d**–**f** Dissected, alizarin red-/alcian blue-stained neurocrania of CA (**d**), DC (**e**) and F1 (**f**) specimens in dorsal view. *Scale bar* 5 mm. **g**–**i** Individual neurocranial bones labeled on Camera Lucida drawings of CA (**g**), DC (**h**) and F1 (**i**). **j**–**l** Dissected, alizarin red-/alcian blue-stained heads of CA (**j**), DC (**k**) and F1 (**l**) specimens in ventral view. *Scale bar* 5 mm. **m**–**o** Individual neurocranial bones labeled on Camera Lucida drawings of CA (**m**), DC (**n**) and F1 (**o**). ***p < 0.001, Tukey’s HSD test. Bone nomenclature after [64]. *BO* basioccipital, *EC* ethmoid cartilage, *EO* epiotic, *EOC* exoccipital, *FR* frontal, *LE* lateral ethmoid, *ME* mesethmoid, *PA* parietal, *PO* pterotic, *PRO* prootic, *PS* parasphenoid, *SO* sphenotic, *SOC* supraoccipital, *VO* vomer

To identify the basis of head shape differences, neurocrania of CA, DC and F1 hybrid adult specimens were dissected and examined in their lateral (Fig. 3), dorsal and ventral aspects (Fig. 4). Canonical variate analysis of lateral landmarks 9–12 and 16 found one axis of significant divergence (Wilk’s *λ* = 0.0371, *χ*^2^ = 72.4799, *df* = 6, *p* = 1.26617e−13). Vectors reflecting CV1 variation around the CA landmark configuration supported a general difference in neurocranium aspect ratio (Fig. 3b), with a more pronounced deformation of preorbital landmarks 9, 12 and 16. This more detailed morphometric analysis reveals that expansion of the preorbital region in DC results from A-P elongation and reorientation of the vomer (VO) and lateral ethmoid (LE) in combination with a pronounced positional shift of the mesethmoid (ME; arrows and asterisks in Fig. 3h–j).

In lateral view, with the landmark configurations from all specimens backtransformed for a SL of 10 cm, preorbital length is 91.64 % longer in DC as measured by the distance between landmarks 9 and 16 (Fig. 3d; Table 1). The F1 values for this measurement are closer to those of DC than of CA. Directly posterior to the preorbital bones, above and below the eye, the frontal (FR) and parasphenoid (PS) bones also differ markedly in size along the A–P axis between CA and DC, and there is a pronounced difference in PS orientation as well (Fig. 3e, f, h, i).

In dorsal view, with the landmark configurations from all specimens backtransformed for a SL of 10 cm, the neurocranium is 46.25 % longer in DC (as measured between landmarks 1 and 4) and 67.47 % wider in CA (as measured between landmarks 3 and 5; Fig. 4a–c; Table 1). Dorsal and ventral measurements revealed that shape differences in the following bones contributed to the greater neurocranial length of DC: VO, LE, ME, FR and PS (Fig. 4d–o). Most of these bones are located in the preorbital region (VO, LE, ME; asterisks in Fig. 4g–i, m–o). In contrast, differences in neurocranial width were most pronounced in the postorbital region in DC, with noticeable medio-lateral (M-L) narrowing of bones ventral to the otic cartilage: sphenotic (SO) and pterotic (PO; Fig. 4d–o).

Dorsal and ventral measurements of F1 neurocrania suggest that DC alleles influencing preorbital and neurocranial length tend to be dominant over those of CA (Fig. 4b), while alleles of both species have roughly codominant influence on neurocranial width (Fig. 4c).

#### Jaws

**Fig. 5:**
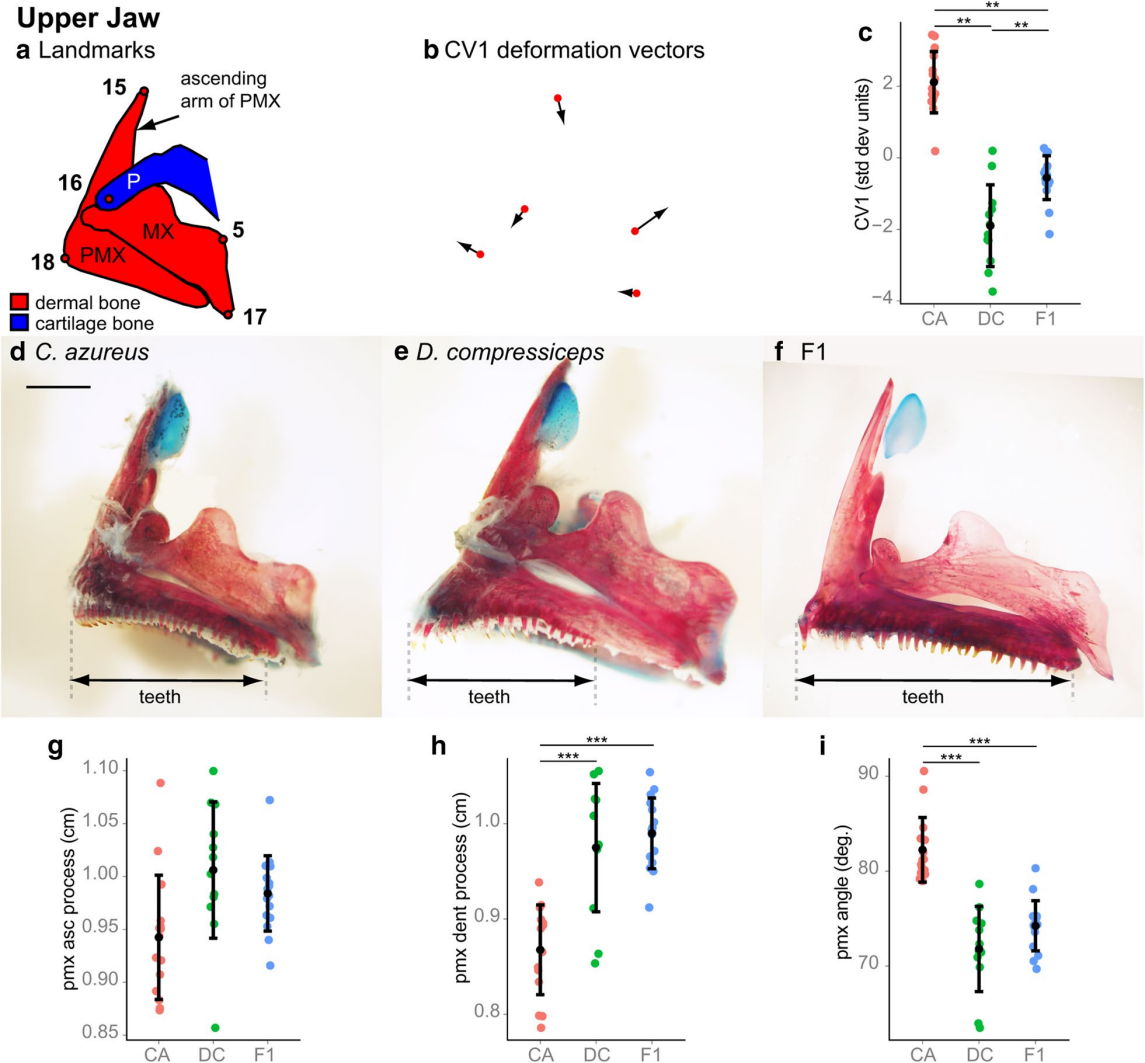
Shape and size differences between the upper jaw skeletons of CA, DC and F1 hybrids. **a** Camera Lucida drawing of CA upper jaw indicating anatomical landmarks. **b** CV1 deformation vectors on CA landmark configuration. **c** Mean and distribution of CA (*n* = 15), DC (*n* = 12) and F1 (*n* = 16) individuals along the CV1 axis, expressed in within-species standard deviation units. **p < 0.001, Bartlett’s test. **d**–**f** Dissected, alizarin red-/alcian blue-stained upper jaw skeletons of CA (**d**), DC (**e**) and F1 (**f**) specimens. *Scale bar* 2.5 mm. **g**–**i** Mean and distribution of the CA, DC and F1 premaxillary ascending process length (**g**), premaxillary dentigerous process length (**h**) and premaxillary process angle (**i**). ***p < 0.001, Tukey’s HSD test. Bone nomenclature after [64]. *MX* maxilla, *P* palatine, *PMX* premaxilla

**Fig. 6:**
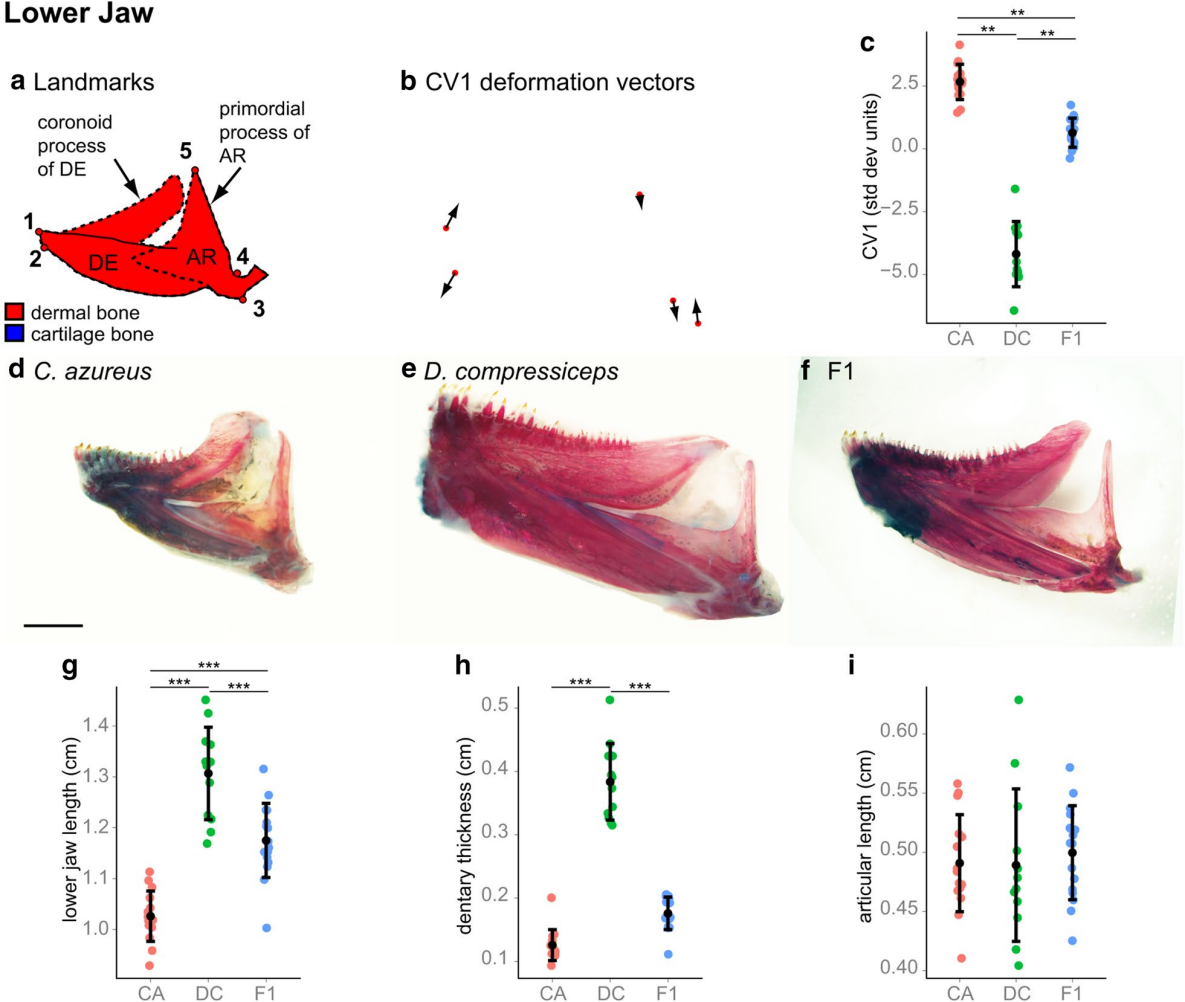
Shape and size differences between the lower jaw skeletons of CA, DC and F1 hybrids. **a** Camera Lucida drawing of CA lower jaw indicating anatomical landmarks. *Dotted lines* indicate approximate locations of bone boundaries. **b** CV1 deformation vectors on CA landmark configuration. **c** Mean and distribution of CA (*n* = 15), DC (*n* = 12) and F1 (*n* = 16) individuals along the CV1 axis, expressed in within-species standard deviation units. **p < 0.001, Bartlett’s test. **d**–**f** Dissected, alizarin red-/alcian blue-stained lower jaw skeletons of CA (**d**), DC (**e**) and F1 (**f**) specimens. *Scale bar* 2.5 mm. **g**–**i** Mean and distribution of CA, DC and F1 lower jaw length (**g**), dentary thickness (**h**) and articular process length (**i**). ***p < 0.001, Tukey’s HSD test. Bone nomenclature after [64]. *AR* articular, *DE* dentary

The two parental species display strong differences in jaw size and shape (Figs. 2, 5, 6 [5]). The upper jaws are composed of two bones: the tooth-bearing premaxilla (PMX) and the maxilla (MX; Fig. 5a). Canonical variate analysis of lateral landmarks 5 and 15–18 identified one significantly different CV axis (Wilk’s *λ* = 0.1530, *χ*^2^ = 70.3949, *df* = 12, *p* = 2.7017e−10) (Fig. 5b, c). CV1 deformation vectors revealed three upper jaw shape differences, all of which were associated with PMX anatomy: (1) length of the ascending arm; (2) length of the dentigerous (tooth-bearing) arm; and (3) the angle between these two processes (anatomy sensu Barel et al. 1976) [40]. PMX ascending arm length (distance between lateral landmarks 15 and 18) was 6.75 % longer in DC, but this difference was not significant (Tukey’s HSD; Fig. 5g; Table 1). PMX dentigerous arm length (as estimated by the distance between lateral landmarks 17 and 18) was significantly greater in DC (12.37 %; Tukey’s HSD; Fig. 5h; Table 1). A 10.4° reduction in the angle between these processes in DC was also significant (Tukey’s HSD; Fig. 5i; Table 1). Interestingly, F1 hybrid PMX process lengths and angles were very similar to those of DC (Fig. 5f–i; Table 1), which indicates a clear dominance of DC alleles over CA alleles in determining PMX morphology. Conversely, F1 tooth coverage of the PMX suggests dominance of CA alleles. Teeth cover the entire A-P extent of the PMX dentigerous arm in CA and the F1 progeny, but only cover roughly the anterior two-thirds of its length in DC (Fig. 5d–f).

The lower jaws are composed of two major bones: the tooth-bearing dentary (DE) and the articular (AR; Fig. 6a). Canonical variate analysis of lateral landmarks 1–5 identified a single axis of significant difference between CA and DC (Wilk’s *λ* = 0.0516, *χ*^2^ = 111.1926, *df* = 12, *p* < 2.22045e−16). CV1 deformations of CA landmarks revealed three lower jaw shape differences: (1) lower jaw length (distance between landmarks 2 and 3); (2) DE thickness (distance between landmarks 1 and 2); and (3) AR primordial process length (distance between landmarks 4 and 5). There were significant differences in lower jaw length (27.4 % longer in DC) and DE depth (201.8 % thicker in DC), but not in AR length (only 0.35 % longer in DC; Fig. 6; Tukey’s HDS; Table 1). Lower jaw shape and size in F1 hybrids indicate rough allelic codominance for lower jaw length and dominance of CA alleles for DE depth.

#### Suspensorium and opercular series

**Fig. 7:**
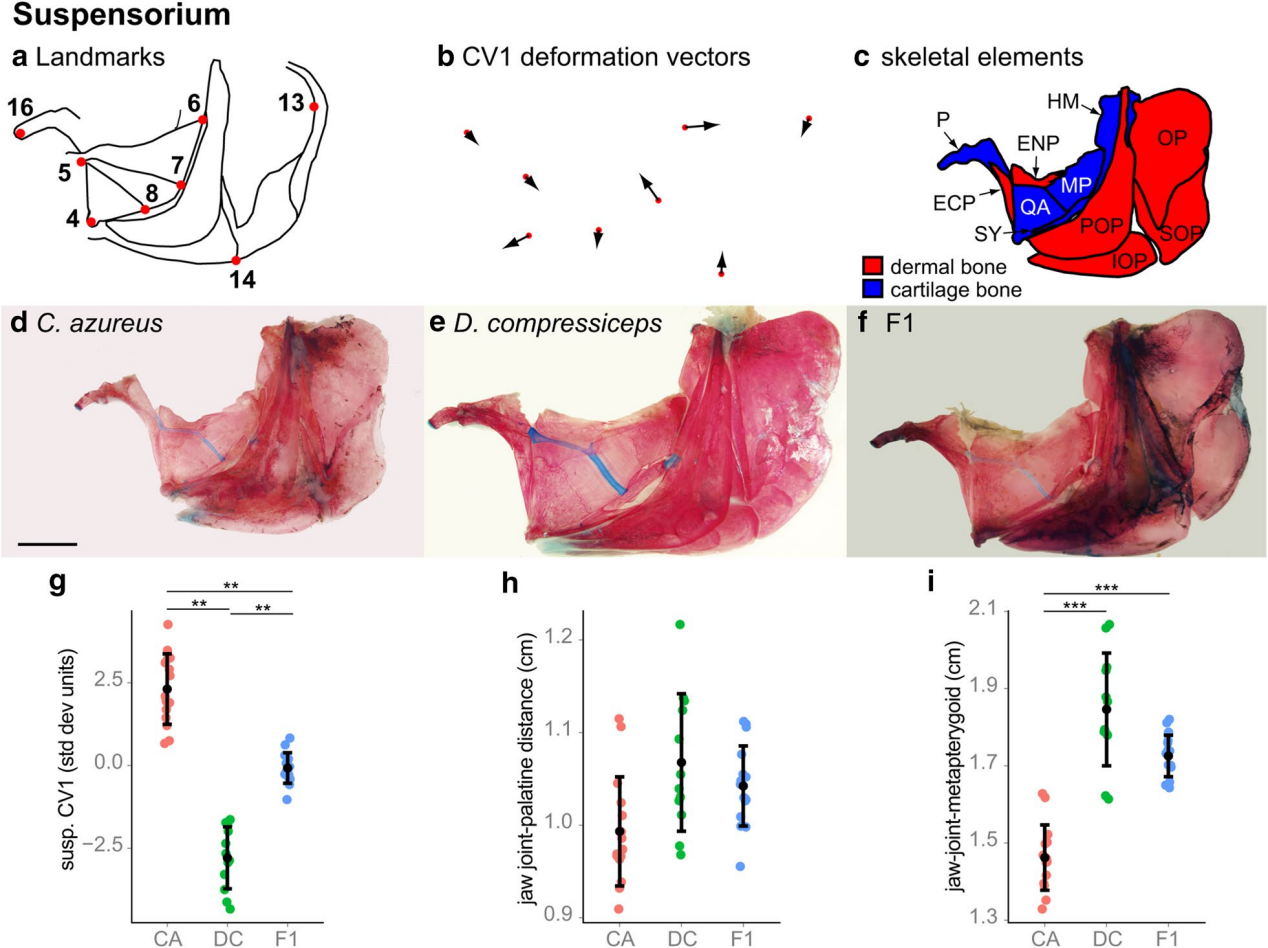
Shape and size differences between the suspensorium and opercular series skeletons of CA, DC and F1 hybrids. **a** Camera Lucida drawing of CA suspensorium and opercular series indicating anatomical landmarks. **b** CV1 deformation vectors on CA landmark configuration. **c** Camera Lucida drawing of CA suspensorium and opercular series bones. **d**–**f** Dissected, alizarin red-/alcian blue-stained suspensorium and opercular series skeletons of CA (**d**), DC (**e**) and F1 (**f**) specimens. *Scale bar* 5 mm. **g** Mean and distribution of CA (*n* = 15), DC (*n* = 12) and F1 (*n* = 16) along the CV1 axis, expressed in within-species standard deviation units. **p < 0.001, Bartlett’s test. **h**, **i** Mean and distribution of CA (*n* = 15), DC (*n* = 12) and F1 (*n* = 16) jaw joint-palatine distance (**h**) and jaw joint-metapterygoid distance (**i**). ***p < 0.001, Tukey’s HSD test. Bone nomenclature after [64]. *ECP* ectopterygoid, *ENP* entopterygoid, *HM* hyomandibular, *IOP* interoperculum, *MP* metapterygoid, *OP* operculum, *P* palatine, *POP* preoperculum, *QA* quadrate, *SOP* suboperculum, *SY* symplectic

The suspensorium articulates with the upper and lower jaws anteriorly, with the neurocranium posterio-dorsally and with the opercular series posteriorly. It contains the oral jaw biting muscles (the adductor mandibulae; see Fig. 1) and serves important functional roles in both feeding and ventilation. The suspensorium of DC is much elongated in comparison with CA (Figs. 1, 7). Canonical variate analysis of lateral landmarks 4–8, 13, 14 and 16 identified a single axis of significant difference between CA and DC (Wilk’s *λ* = 0.1168, *χ*^2^ = 40.7961, *df* = 12 *p* = 5.29996e−05) (Fig. 7g). CV1 deformations of CA landmarks were strongest for landmarks 4, 6 and 7 (Fig. 7b), which indicates differences in the size of the quadrate (QA), metapterygoid (MP), symplectic (SY) and hyomandibular (HM) bones. There was a significant difference in posterior suspensorium depth (distance between landmarks 4 and 6) between CA and DC (26.3 % longer in DC; Tukey’s HSD; Fig. 7i; Table 1), but no significant difference in anterior suspensorium depth (distance between lateral landmarks 4 and 16) between CA and DC (only 7.50 % longer in DC; Tukey’s HSD; Fig. 7h; Table 1). F1 hybrid suspensorium depth was very similar to that of DC (Fig. 7i), which suggests a dominance of DC alleles.

**Fig. 8:**
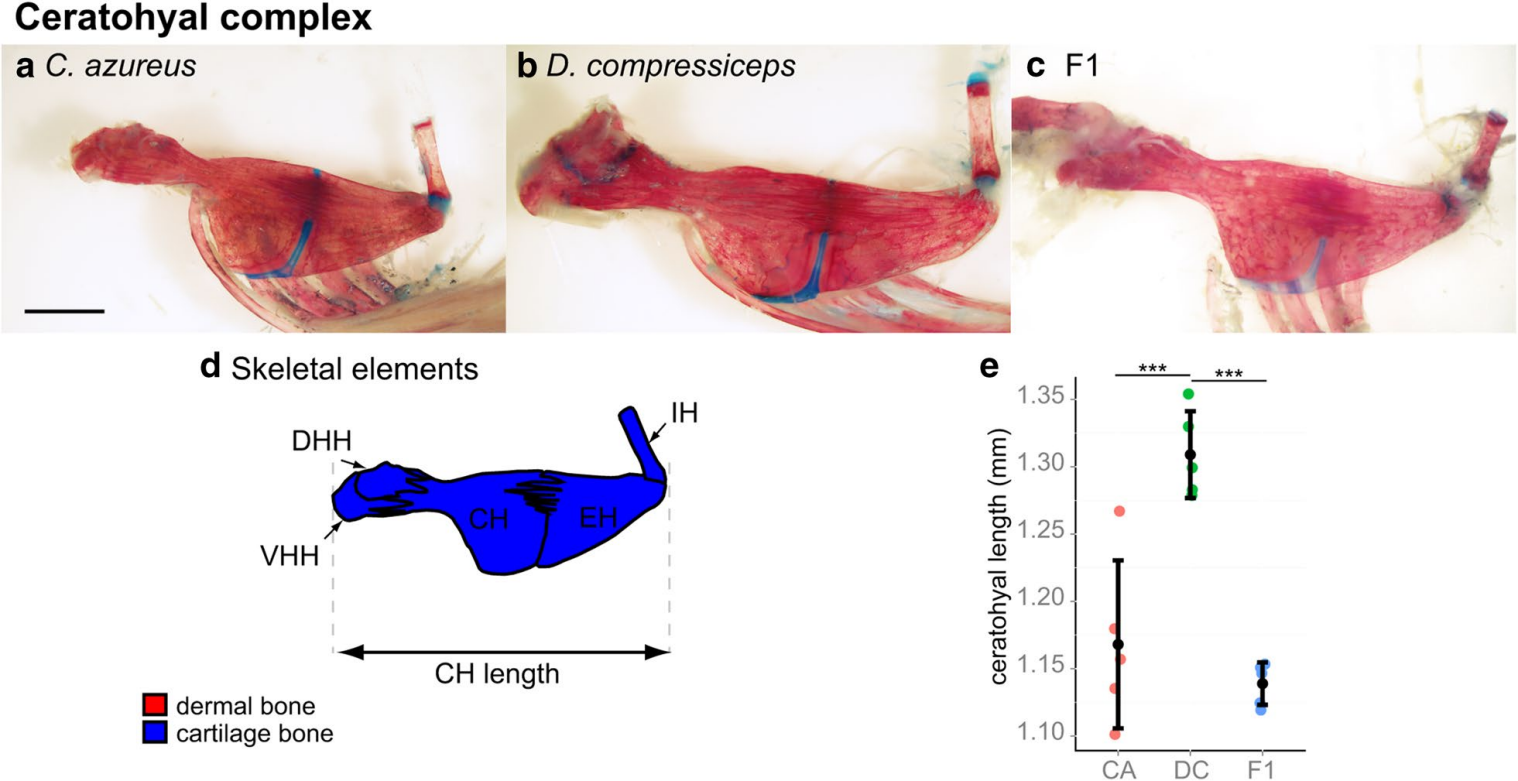
Size differences between ceratohyal complex skeletons of CA, DC and F1 hybrids. **a**–**c** Dissected, alizarin red-/alcian blue-stained ceratohyal complex skeletons of CA (**a**), DC (**b**) and F1 (**c**) specimens. *Scale bar* 2.5 mm. **d** Camera Lucida drawing of CA ceratohyal complex bones. **e** Mean and distribution of CA (*n* = 5), DC (*n* = 5) and F1 (*n* = 5) ceratohyal length. ***p < 0.001, Tukey’s HSD test. Bone nomenclature after [64]. *CH* ceratohyal, *DHH* dorsal hypohyal, *EH* epihyal, *IH* interhyal, *VHH* ventral hypohyal

Each suspensorium connects ventrally to a ceratohyal complex, which bears the ventral-most bones of the opercular series: the branchiostegal rays (Fig. 8). The ceratohyal complex was not included in our whole-head morphometric analysis due to its internal location, but its length was measured and found to be significantly longer (12.1 %; Tukey’s HSD; Table 1) in DC than in CA (Fig. 8d, e). F1 hybrid ceratohyal complex length recapitulated that of CA, indicating dominance of CA alleles on this character (Fig. 8e).

#### Gill skeleton and pharyngeal jaws

**Fig. 9:**
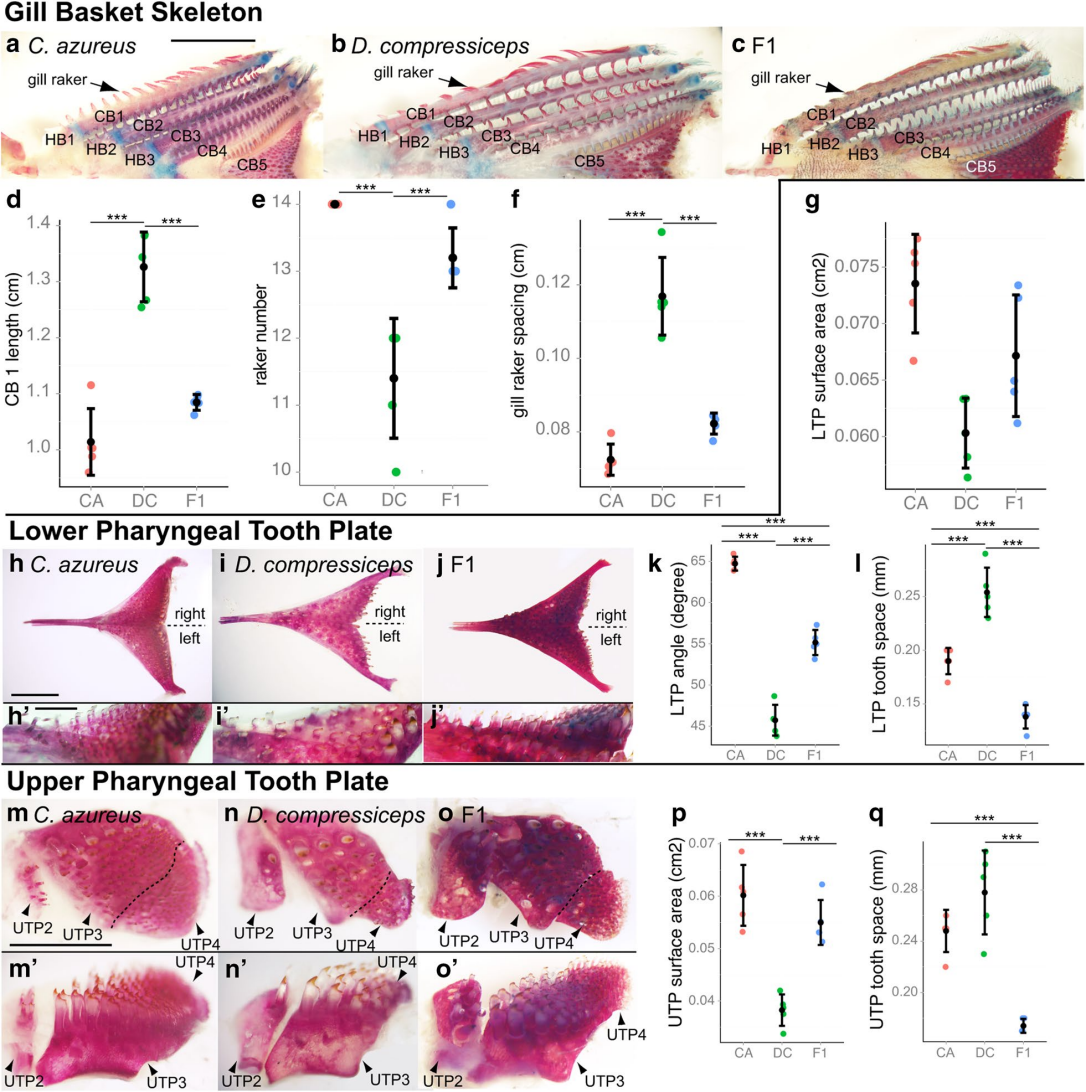
Size differences between the gill arch skeletons of CA, DC and F1 hybrids. **a**–**c** Dissected, alizarin red-/alcian blue-stained gill arch skeletons of CA (**a**), DC (**b**) and F1 (**c**) specimens. *Scale bar* 5 mm. **d**–**f** Mean and distribution of CA, DC and F1 CB1 length (**d**), gill raker number (**e**) and gill raker spacing (**f**) (*n* = 5, each). **h**–**j** Dissected, alizarin red-/alcian blue-stained lower tooth plates of CA (**h**, **h**′), DC (**i**, **i**′) and F1 (**j**, **j**′) specimens. *Scale bar* 2.5 mm (**h**–**j**) and 1.0 mm (**h**′, **i**′, **j**′). **g**, **k**, **l** Mean and distribution of CA, DC and F1 lower tooth plate surface area (**g**), angle (**k**) and tooth spacing (**l**) (*n* = 5, each). **m**–**o** Dissected, alizarin red-/alcian blue-stained upper tooth plates of CA (**m**, **m**′), DC (**n**, **n**′) and F1 (**o**, **o**′) specimens. *Scale bar* 2.5 mm. **p**, **q** Mean and distribution of CA, DC and F1 upper tooth plate surface area (**p**) and tooth spacing (**q**) (*n* = 5, each). ***p < 0.001, Tukey’s HSD test. Bone nomenclature after [64]. *CB* ceratobranchial, *HB* hypobranchial, *LTP* lower tooth plate, *UTP* upper tooth plate

The gill skeleton determines the shape and size of the pharyngeal cavity, supports the gills, filters food particles and processes swallowed food. The overall differences in head size, length, width and diet between CA and DC led us to predict that the piscivorous DC would have a larger gill skeleton that would allow it to process large prey. Here again, we did not include the gill skeleton in our morphometric analysis due to its internal location. Instead, we measured the length of the first ceratobranchial bone (CB1) as a proxy for all CB elements (Fig. 9a–c) and found it to be 30.8 % longer in DC than in CA (Fig. 9d). Planktivorous fish also tend to have smaller, more tightly spaced gill rakers than piscivores, which function to capture small food particles before they exit the gills. CA individuals have more gill rakers on CB1 (14 versus 11.4; Fig. 9e; Table 1), which is shorter than in DC, effectively resulting in a significant difference in raker spacing between the two species (0.7 mm versus 1.2 mm; Tukey’s HSD; Fig. 9f; Table 1). F1 hybrid CB1 length, raker number (13.2) and raker spacing (0.8 mm) are more similar to CA (Fig. 9d–f; Table 1), which suggests dominance of CA alleles over DC alleles.

Cichlid fishes have dorsal and ventral pharyngeal tooth plates that are used for processing food and transporting it into the gut. The lower tooth plate is ossified over the CB5 element and fused along its midline (Fig. 9h–j). There were significant differences in lower tooth plate surface area, the angle formed by lower tooth plate arms and tooth spacing (Fig. 9g, k, l; Tukey’s HSD; Table 1): DC has 18 % smaller (surface area), narrower plates (19° smaller angle) with more widely spaced teeth (253 um in DC versus 187 um in CA) than CA, and F1 hybrid phenotypes suggest codominance of DC and CA alleles on lower tooth plate size and shape. However, F1 hybrid tooth spacing is transgressive—141 um (outside the range established by CA and DC), indicative of synergistic activities between CA and DC alleles. Similar differences in skeletal size and tooth density were observed in upper tooth plates (UTPs; Fig. 9m–o)—DC has 36.4 % smaller UTP3 + 4 elements with more widely spaced teeth (279 um in DC versus 247 um in CA; Fig. 9p, q; Table 1). In this case, CA alleles seem dominant over DC alleles in their effects on UTP3 + 4 size, while synergistic effects of DC and CA alleles on tooth spacing are suggested by a transgressive F1 hybrid phenotype (172 um).

#### Endochondral growth zones and dermal bone sutures

**Fig. 10:**
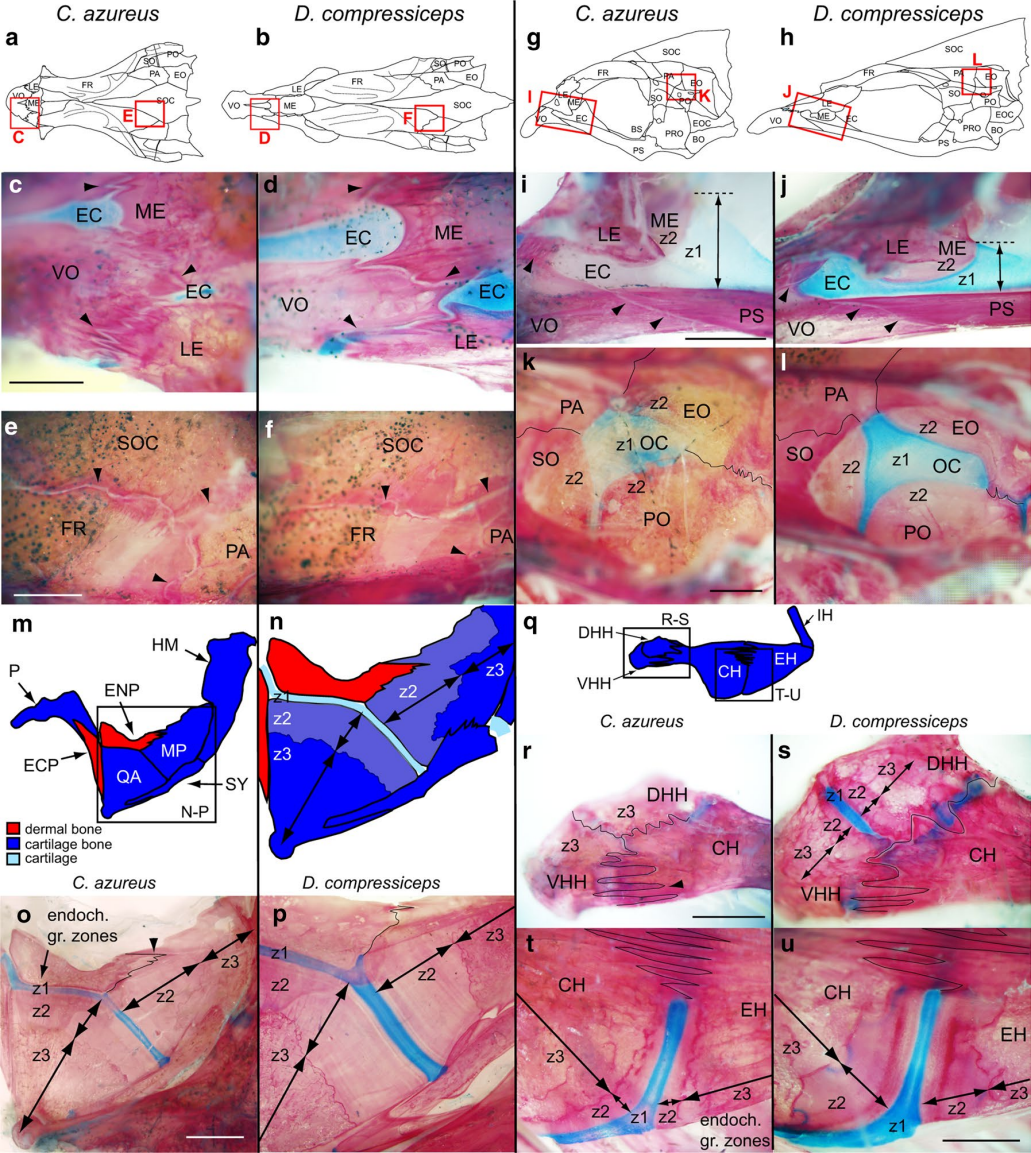
Shape and size differences in skeletal growth zones between CA and DC. **a**, **b** Camera lucida drawings of the dorsal aspect of CA (**a**) and DC (**b**) neurocrania with *boxed areas* highlighting suture regions shown in (**c**–**f**). **c**, **d** Dorso-lateral aspect of preorbital region showing sutures (*arrowheads*) between VO, LE and ME bones in CA and DC. **e**, **f** Dorso-lateral aspect of cranial vault region showing sutures (*arrowheads*) between FR, PA and SOC bones in CA and DC. *Scale bar* 100 μm. **g**, **h** Camera lucida drawings of the lateral aspect of CA (**g**) and DC (**h**) neurocrania with boxed areas highlighting suture regions shown in **i**–**l**. **i**, **j** Lateral aspect of preorbital region showing EC and sutures (*arrowheads*) between VO, LE and PS bones, and endochondral growth zone between EC and ME in CA and DC. *Z1*–*2* zone 1–2 (**k**, **l**) dorso-lateral aspect of otic region showing sutures (*lines*) between EO, PO, SO and PA, and endochondral growth zone between OC and EO, PO and SO in CA and DC. *Scale bar* 100 μm. *Z1*–*2* zone 1–2 (**m**, **n**) Camera lucida drawings of the suspensorium in CA (**m**) and endochondral growth zone between QA and MP (**n**). **o**, **p** Endochondral growth zone between QA and MP in CA (**o**) and DC (**p**). *Scale bar* 200 μm. *Z1*–*3* zone 1–3 (**q**) Camera Lucida drawing of the ceratohyal complex skeleton in CA with boxed areas highlighting endochondral growth zones shown in **r**–**u**. **r**, **s** Endochondral growth zones and sutures (*lines*) between CH, DHH and VHH in CA (**r**) and DC (**s**). **t**, **u** Endochondral growth zones and sutures (*lines*) between CH and EH in CA (**t**) and DC (**u**). *Scale bar* 100 μm. *Z1*–*3* zone 1–3. Bone nomenclature after [64]. *DHH* dorsal hypohyal, *EC* ethmoid cartilage, *ECP* ectopterygoid, *ENP* entopterygoid, *EO* epiotic, *FR* frontal, *HM* hyomandibular, *LE* lateral ethmoid, *ME* mesethmoid, *MP* metapterygoid, *OC* otic cartilage, *P* palatine, *PS* parasphenoid, *PA* parietal, *PO* pterotic, *QA* quadrate, *SO* sphenotic, *SOC* supraoccipital crest, *SY* symplectic, *VHH* ventral hypohyal, *VO* vomer

Skeletal shape and size differences must arise due to differential cartilage/bone growth and remodeling. To gain insight into species-specific differences in growth, we examined sutures, which are well-characterized regions of growth in dermal bones, as well as cartilage growth zones of endochondral bones [13, 19, 41]. We compared the shape and size of sutures and endochondral growth zones in skeletal elements of the neurocranium and suspensorium that differ between CA and DC: the VO, LE, ME and PS (Fig. 10a–d, g–j); the FR, PA and SOC (Fig. 10e, f); the PA, EO, PO and SO (Fig. 10k, l); the QA and MP (Fig. 10m–p); the ventral and dorsal HH and CH (Fig. 10q–s); and the CH and EH (Fig. 10t, u). Qualitative observations of suture organization indicated local differences between neurocranial preorbital bones, where suture patterns between the VO, ME and LE were less intricately folded in DC than in CA (Fig. 10c, d, i, j). These differences were not observed in neurocranial vault sutures (Fig. 10e, f).

Three presumptive zones of endochondral growth were observed in our skeletal preparations (Fig. 10i–l, n–u). Based on assumed organizational similarities with long-bone growth plates, we speculate that zone 1 contains resting and proliferative chondrocytes, as indicated by alcian blue staining. Zone 2 contains mature and hypertrophic chondrocytes ensheathed in bone matrix, as indicated by alizarin red staining associated with cellular cartilage. Zone 3 is made of bone and does not contain chondrocytes. Zones 1 and 2 were consistently larger in DC than in CA, at the expense of zone 3, in all elements examined—QA and MP (Fig. 10o, p) as well as CH and EH (Fig. 10t, u). In some instances, the distinction between cartilage- and dermal bone growth zones was blurred, as at the junctions between the dorsal and ventral HH and the CH (Fig. 10r, s), where endochondral growth zones were partly replaced by sutures, and this replacement was more advanced in CA than in DC. In the neurocranium, zones 1 and 2 were also observed at the junction between the ethmoid cartilage (EC) and the ME, as well as between the otic cartilage (OC) and the PA, EO and PO bones, where differences in zone 1–zone 2 junction shape were observed (Fig. 10i–l).

### Developmental basis of divergent head skeleton morphologies

In order to identify the developmental events leading to the formation of the skeletal element shape and size differences observed between the heads of CA and DC, we fixed and stained specimens of each species at developmental stages chosen to demonstrate differences in embryonic cartilage patterning (5 dpf—1CFRE), larval cartilage growth (8 dpf—3CFRE) and/or bone development (15 dpf—9CFRE—11 mm) [42].

#### Neurocranial development

**Fig. 11:**
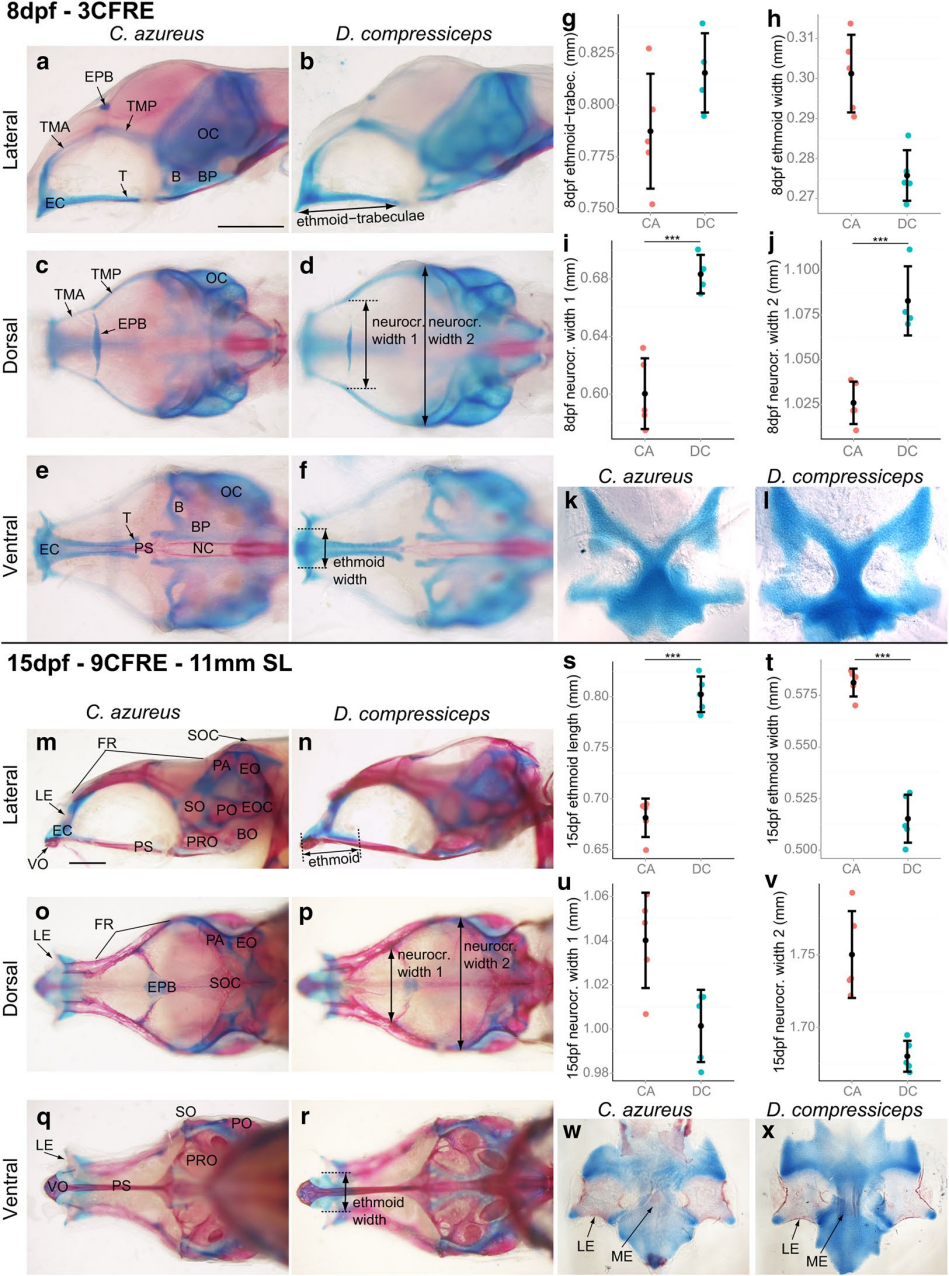
Size differences between the neurocrania of CA and DC larvae. **a**–**f** Lateral (**a**, **b**), dorsal (**c**, **d**) and ventral (**e**, **f**) views of dissected alizarin red-/alcian blue-stained neurocranial skeletons of 8 dpf CA (**a**, **c**, **e**) and DC (**b**, **d**, **f**) larvae. *Scale bar* 0.5 mm. **g**–**j** Mean and distribution of 8 dpf CA and DC (*n* = 5, each) ethmoid–trabeculae length (**g**), ethmoid cartilage width (**h**), neurocranial anterior (**i**) and posterior width (**j**). **k**, **l** Flat-mounted rostral portion of the alcian blue-stained EC in CA (**k**) and DC (**l**), dorsal/top, ventral/bottom. **m**–**r** Lateral (**m**, **n**), dorsal (**o**, **p**) and ventral (**q**, **r**) views of dissected alizarin red-/alcian blue-stained neurocranial skeletons of 15 dpf CA (**m**, **o**, **q**) and DC (**n**, **p**, **r**) larvae. *Scale bar* 0.5 mm. **s**–**v** Mean and distribution of 15 dpf CA and DC (*n* = 5, each) ethmoid cartilage length (**s**) and width (**t**), and neurocranial anterior (**u**) and posterior (**v**) width. ***p < 0.001, Tukey’s HSD test. (**w**, **x**) Flat-mounted rostral portion of the alizarin red-/alcian blue-stained EC in 15 dpf CA (**w**) and DC (**x**), dorsal/top, ventral/bottom. Bone nomenclature after [64]. *B* basicapsular fenestra, *BP* basal plate, *BO* basiotic, *EC* ethmoid cartilage, *EO* epiotic, *EOC* epioccipital, *EPB* epiphyseal bar, *FR* frontal, *LE* lateral ethmoid, *ME* mesethmoid, *NC* notochord, *OC* otic capsule, *PA* parietal, *PO* pterotic, *PRO* prootic, *PS* parasphenoid, *SO* sphenotic, *SOC* supraoccipital crest, *T* trabecula, *TMA* taenia marginalis anterior, *TMP* taenia marginalis posterior, *VO* vomer

The spatially clustered morphological differences between the adult neurocrania of CA and DC in the preorbital and otic regions suggest that ontogenic differences affecting the cartilage precursors from which adult bones are derived may be responsible for these differences. For instance, differences in ethmoid cartilage (EC) embryonic patterning are predicted to affect the shape and size of all bones that derive from or form in close association with this cartilage. Bones derived from the EC are the ME and the LEs, while the VO is a dermal bone that differentiates directly ventral to and partially surrounds the EC (Fig. 11m–r). Bones derived from the embryonic otic capsule are the EO, SO, PO and EOC (Fig. 11k–p).

The EC first differentiates as a plate in between and extending anterior to the rod-like trabeculae (Fig. 11a–f, k, l). Measurements of ethmoid–trabecular size revealed slight differences in length and width at 8 dpf between CA and DC, but these were not significant (Fig. 11e–h, k, l; Tukey’s HSD; Table 2). Differences in neurocranial width also became noticeable by 8 dpf, but in an unexpected manner, as the head was significantly wider in DC (11.1 % wider at the epiphyseal bar and 5.4 % wider posteriorly; Tukey’s HSD; Table 2) than that in CA at this stage (Fig. 11i, j).

**Table 2:** Embryo- and larva measurements and statistics

	CA	DC	DC value as % of CA	Tukey’s HST *p* value	*p* < 0.001
Neurocranium (mm)					
8 dpf					
EC + trabeculae length	0.787	0.8	101.6	0.1293217	
EC width	0.301	0.276	91.5	0.0011728	
NC width 1—anterior	0.6	0.667	111.1	0.0005305	***
NC width 2—posterior	1.026	1.081	105.4	0.0009214	***
15 dpf					
EC length	0.681	0.802	117.8	5.60E−06	***
EC width	0.581	0.515	88.6	4.20E−06	***
NC width—anterior	1.04	1	103.9	0.0126731	
NC width—posterior	1.75	1.68	96	0.0011456	
*Pharyngeal arches*					
Linear measurements (mm)					
15 dpf					
PMX angle (deg.)	87.794	81.675	93.0	1.37E−05	***
PMX asc. proc. length	0.3644	0.4203	115.3	3.23E−05	***
PMX tooth. proc. length	0.4065	0.4399	108.2	0.0004156	***
DE thickness	0.0636	0.099	155.7	1.20E−06	***
LJ length	0.6788	0.6907	101.8	0.1285571	
PQ anterior length	0.6444	0.6718	104.2	0.1025203	
PQ posterior length	0.6282	0.7539	120.0	2.00E−07	***
CH length	0.6333	0.6702	105.8	0.0008139	***
CB1 length	0.5623	0.5745	102.2	0.1286012	
Raker number	14	10	71.4	0	***
Raker spacing	2.5E−05	3.7E−05	147.6	0	***
LTP angle (deg.)	88.712	93.188	105.0	0.0035458	
Growth zone width (um)					
PQ	106.78	137.72	129.0	0	***
HS	120.09	131.25	109.3	0.006035	
CH	42.45	39.95	94.1	0.9428638	
Surface area measurements (mm^2^)					
5 dpf					
MC	0.0317	0.0327	103.3	0.9955359	
PQ	0.0564	0.0656	116.5	0.3564464	
HS	0.0674	0.0756	112.1	0.0444626	
CH	0.0471	0.0487	103.4	0.9974972	
CB1	0.0203	0.0198	97.2	0.9995432	
8 dpf					
MC	0.0424	0.0491	115.8	0.0384713	
PQ	0.0848	0.1190	140.3	0.0000014	***
HS	0.1046	0.1239	118.5	0.0000015	***
CH	0.0760	0.0767	100.9	0.9999659	
CB1	0.0298	0.0298	100.1	1	
15 dpf					
MC	0.1075	0.1138	105.8	0.0590679	
PQ	0.3013	0.4429	147.0	0	***
HS	0.2760	0.3124	113.2	0	***
CH	0.2476	0.2717	109.7	0.000007	***
CB1	0.0715	0.0821	114.8	0.0006013	***
UTP	0.1013	0.1256	124.0	7.20E−06	***
LTP	0.0907	0.1036	114.2	0.0008328	***

By 15 dpf the EC was 17.8 % longer and 11.3 % narrower in DC (Fig. 11s, t; Tukey’s HSD; Table 2), and the LE and ME had started to ossify (Fig. 11m–r; w–x). The VO was also ossified ventral to the EC. Differences in neurocranial width at this stage started to reflect the adult trend, with the CA neurocranium 4 % wider than that of DC (Fig. 11u, v). At this stage, the frontal bone makes up most of the neurocranial roof and develops as a membrane bone anterior and posterior to the epiphyseal bar cartilage (Fig. 11m–r). No difference in FR bone length was found at this stage (data not shown). The PS was also ossified, but showed no difference in size (data not shown, Fig. 11m, n, q, r). Five bones associated with the otic cartilage were ossified by 15 dpf: the PA, EO, SO, PO and EOC, but no differences were observed between these bones in CA versus DC (Fig. 11m, n).

Overall, a difference in EC length is detectable by 8 dpf and amplified by 15 dpf, supporting the hypothesis that this initial difference may underlie the evolutionary divergence of ME, LE and VO adult preorbital morphologies in CA and DC. In contrast, differences in otic cartilage morphology were not detected at the stages examined and must arise later as the bones grow and remodel.

#### Interspecific developmental differences of first and second arch derivatives

**Fig. 12:**
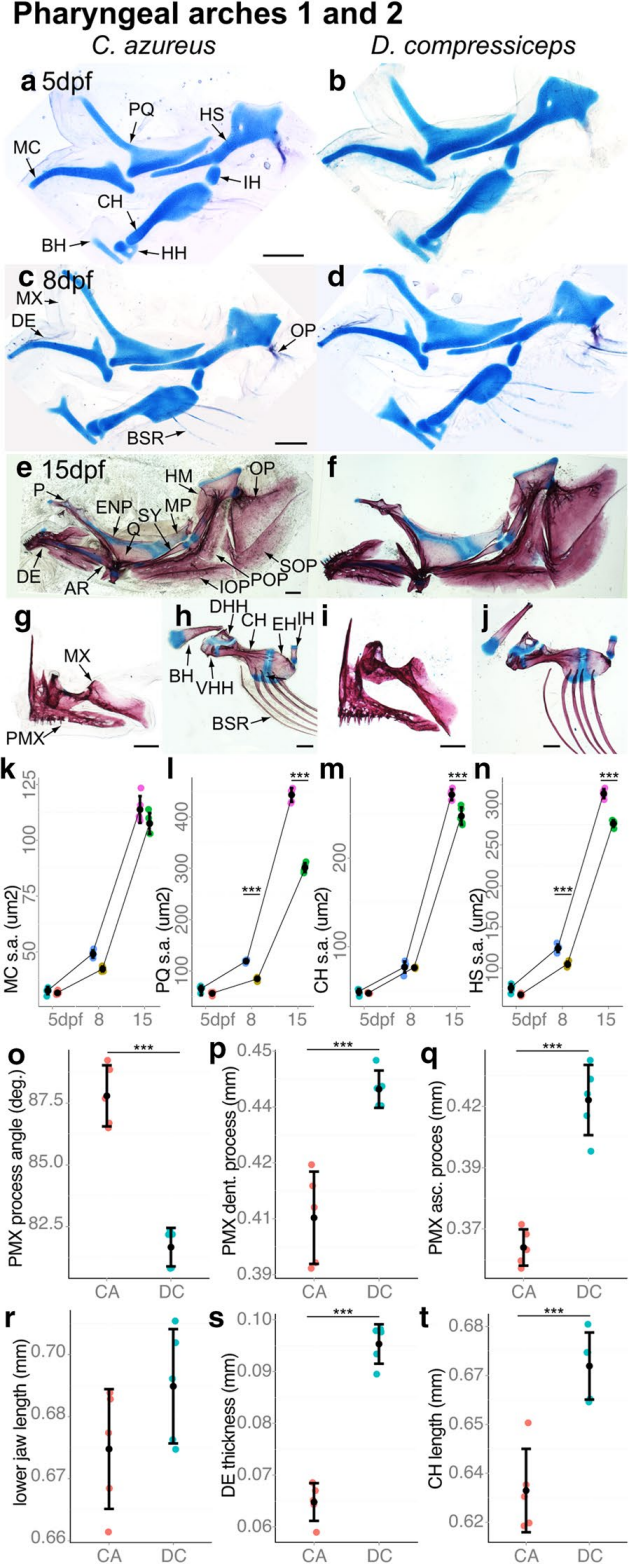
Size differences in skeletal derivatives of pharyngeal arches 1 and 2 between CA and DC larvae. **a**–**f** Dissected and flat-mounted alizarin red-/alcian blue-stained first and second pharyngeal arch skeletons of 5 dpf (**a**, **b**), 8 dpf (**c**, **d**) and 15 dpf (**e**, **f**) CA and DC larvae. **g** Upper jaw skeleton of 15 dpf CA. **h** ceratohyal complex skeleton of 15 dpf CA. **i** Upper jaw skeleton of 15 dpf DC. **j** ceratohyal complex skeleton of 15 dpf DC. **k**–**n** Mean and distribution of MC (**k**), PQ (**l**), CH (**m**) and HS (**n**) surface area in 15 dpf CA and DC (*n* = 5, each). **o**–**q** Mean and distribution of premaxillary processes angle (**o**), dentigerous process length (**p**) and ascending process length (**q**) in 15 dpf CA and DC (*n* = 5, each). **r**–**t** Mean and distribution of lower jaw length (**r**), DE thickness (**s**) and CH length (**t**) in 15 dpf CA and DC (*n* = 5, each). *Scale bar* 200 μm. ***p < 0.001, Tukey’s HSD test. Bone nomenclature after [64]. *AR* articular, *BH* basihyal, *BSR* branchiostegal rays, *CH* ceratohyal, *DE* dentary, *DHH* dorsal hypohyal, *EH* epihyal, *ENP* entopterygoid, *HH* hypohyal, *HS* hyosymplectic, *IH* interhyal, *IOP* interoperculum, *MP* metapterygoid, *MX* maxilla, *OP* operculum, *P* palatine, *PMX* premaxilla, *POP* preoperculum, *Q* quadrate, *SOP* suboperculum, *SY* symplectic, *VHH* ventral hypohyal

As with the neurocranium, the localized morphological differences observed between the adult jaw and suspensorial skeletons of CA and DC suggest that ontogenic differences in a few cartilage precursors may underlie shape and size differences in all of their derivatives. Namely, the concerted differences in QA, MP, SY and HM bone sizes between DC and CA may be initiated by size differences in their precursor cartilage: the palatoquadrate (PQ) and the hyosymplectic (HS; Fig. 12a, b), which are derived from the first and second pharyngeal arch dorsal modules, respectively. Strikingly, PQ size (surface area) was already 16.5 % larger in DC than in CA at 5 dpf, 40.3 % larger at 8 dpf and 47 % larger at 15 dpf (Fig. 12a–f, L; Tukey’s HSD; Table 2). HS was also larger in DC, although to a lesser extent—12.1 % at 5 dpf, 18.5 % at 8 dpf and 13.2 % at 15 dpf (Fig. 12a–f, n; Tukey’s HSD; Table 2). In contrast, Meckel’s (MC) and the ceratohyal (CH) were only 5.8 % and 9.7 % larger in DC at 15 dpf (Fig. 12a–f, k, m; Tukey’s HSD; Table 2). These results suggest that specific difference(s) in PQ and HS cartilage development underlie, at least in part, the differences in size and shape of their bone derivatives in adult DC and CA.

Unlike cartilage-derived bones, groups of dermal bones are not derived from common anlage, although they may be influenced by common developmental signals, such as endothelin-1 on the opercular series [43]. The lower jaw skeleton represents an interesting case in which there is a cartilaginous lower jaw (Meckel’s cartilage) in the embryos and young larvae of teleosts, but where the two bones of the adult lower jaw (DE and retroarticular) do not result from endochondral ossification of this structure. They instead develop entirely or almost entirely from dermal ossifications that form around Meckel’s cartilage (there may be some limited endochondral ossification around the articular–quadrate joint), which is retained within the resulting bony envelope (Fig. 12e, f). Cranial dermal bones associated with the jaws stained with alcian/alizarin (Fig. 12e–j) revealed a subset of the differences at 15 dpf noted in upper and lower jaw dermal bones at adult stages: The PMX angle was 6.1° smaller and the DE already had 70.3 % greater depth in DC (Fig. 12o, s; Tukey’s HSD; Table 2). PMX dentigerous and ascending arms were 10.3 % and 11.56 % longer, respectively, in DC (Fig. 12p, q; Tukey’s HSD; Table 2), while DE length was not significantly different.

As exemplified by differences in PQ and HS formation, shape and size differences affecting cartilage bones in the adult pharyngeal skeleton originate in cartilage development during embryonic stages and thus result in morphologically integrated changes among all derived bones. This replicates the pattern seen with endochondral bone development in the preorbital region of the neurocranium, but the same is not true for dermal bone formation. Developmental changes that affect the shape and size of dermal ossifications have much more spatially restricted effects on the pharyngeal skeleton (e.g., the DE in the lower jaw).

#### Interspecific developmental differences of gill arch derivatives

**Fig. 13:**
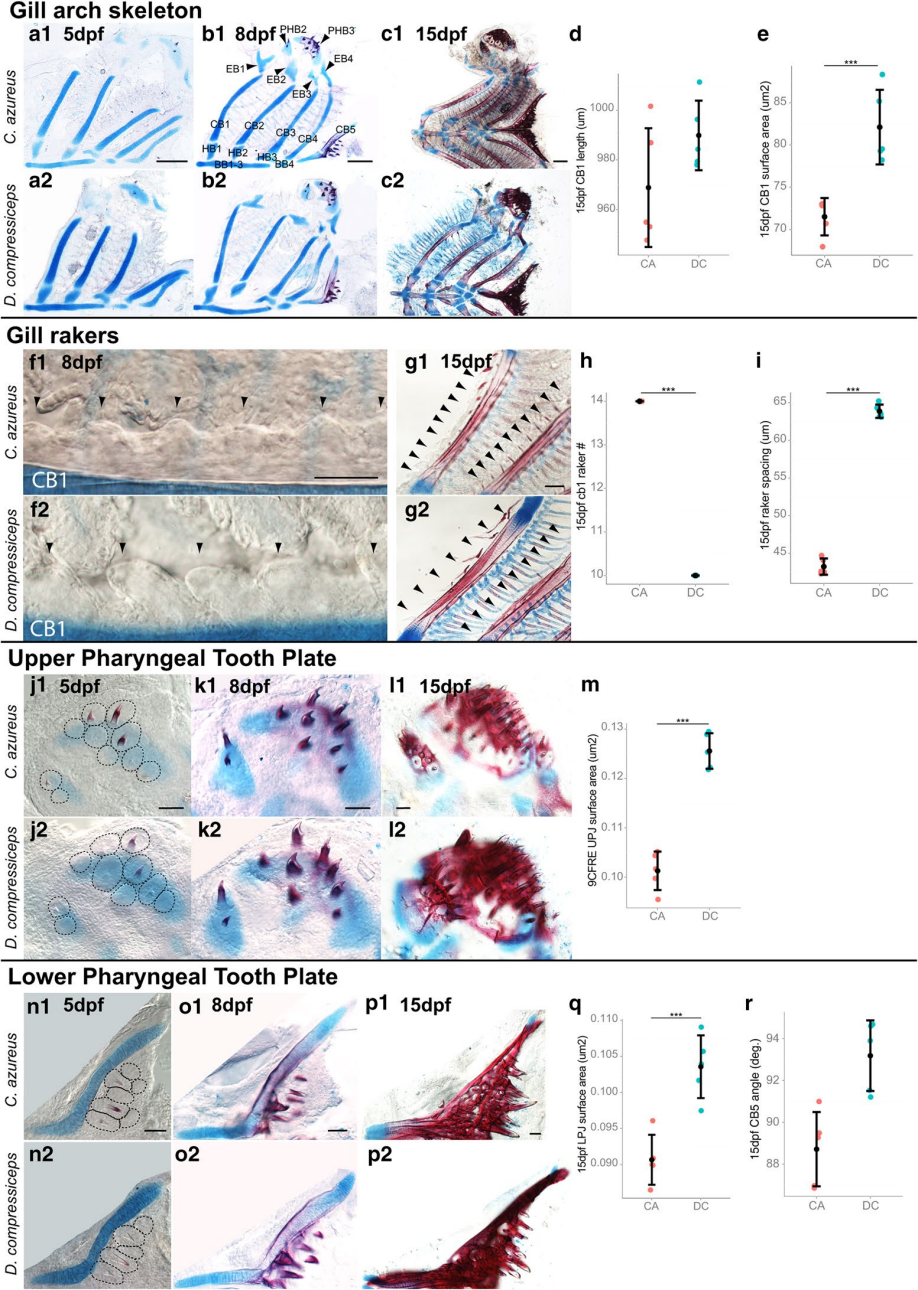
Size differences in skeletal derivatives of the gill arches between CA and DC larvae. **a**–**g** Dissected and flat-mounted alizarin red-/alcian blue-stained gill arch skeletons of 5 dpf (**a1**–**2**), 8 dpf (**b1**–**2**) and 15 dpf (**c1**–**2)** CA and DC larvae. *Scale bar* 200 μm. **d**, **e** Mean and distribution of CB1 length (**d**) and surface area (**e**) in 15 dpf CA and DC (*n* = 5, each). **f1**–**g2** Dissected and flat-mounted alizarin red-/alcian blue-stained CB1 and gill raker buds in 8 dpf (**f1**–**2**) and 15 dpf (**g1**–**2**) CA and DC larvae. *Scale bar* 50 μm. **h**, **i** Mean and distribution of CB1 gill raker number (**h**) and spacing (**i**) in 15 dpf CA and DC (*n* = 5, each). **j1**–**l2** Dissected and flat-mounted alizarin red-/alcian blue-stained upper pharyngeal tooth plate in 5 dpf (**j1**–**2**), 8 dpf (**k1**–**2**) and 15 dpf (**l1**–**2**) CA and DC larvae. **m** Mean and distribution of UTP surface area in 15 dpf CA and DC (*n* = 5, each). **n1**–**p2** Dissected and flat-mounted alizarin red-/alcian blue-stained lower pharyngeal tooth plate in 5 dpf (**n1**–**2**), 8 dpf (**o1**–**2**) and 15 dpf (**p1**–**2**) CA and DC larvae (*n* = 5, each). *Scale bar* 50 μm. **Q**, **R** Mean and distribution of LTP surface area (**q**) and arms angle (**r**) in 15 dpf CA and DC (*n* = 5, each). ***p < 0.001, Tukey’s HSD test. Bone nomenclature after [64]. *BB* basibranchial, *CB* ceratobranchial, *EB* epibranchial, *HB* hypobranchial, *PHB* pharyngobranchial

Interspecific differences in adult gill arch morphology include: (1) CB length; (2) gill raker size and spacing; (3) pharyngeal plate size; and (4) tooth size and spacing. No significant differences in CB length were found at 5, 8 or 15 dpf (Fig. 13d, Tukey’s HSD; Table 2), while CB1 was 19.2 % larger (surface area) in DC at 15 dpf (Fig. 13e; Tukey’s HSD; Table 2), reflecting a difference in thickness. Gill raker buds were observed by 8 dpf in both species (Fig. 13f1, f2) and were already larger and more widely spaced in DC at this stage. By 15 dpf CA had 14 rakers on CB1, while DC had 10 (Fig. 13g1, g2, h), spaced 43.2 um and 63.8 um apart, respectively (Fig. 13i).

Similar to CB development, embryonic development of upper (UTP) and lower pharyngeal tooth plates (LTP) was remarkably similar between CA and DC (Fig. 13j1–k2, n1–o2) in terms of both shape and size, followed by a significant increase in size in DC by 15dpf. UTP and LTP plate sizes were larger in DC (24 % and 14 %, respectively) by 15 dpf (Fig. 13l1–m, p1–q; Tukey’s HSD; Table 2), which did not correlate with adult pharyngeal tooth plate sizes. In addition, the angle between the lateral arms of the LTP was 4.5° larger in DC (Fig. 13p1, p2, r), which suggests that widening of the pharyngeal cavity precedes its narrowing in DC. A similar number of equally spaced tooth buds were observed on the UTP (Fig. 13j1–l2) and LTP (Fig. 13n1–p2) at 5, 8 and 15 dpf in both species, which indicates that differences in tooth number and spacing appear at later stages.

Overall, our data indicate that unlike differences in neurocranial and anterior pharyngeal arch skeletal elements, shape and size differences in the 15 dpf gill arch skeleton do not correlate with adult morphology; DC larvae have thicker CB elements and larger tooth plates than CA larvae. While this morphology may better adapt DC juveniles for the capture of larger prey items, a shift in gill arch developmental trajectory must occur later in life to adopt the adult morphology. In contrast, gill raker number and spacing do correlate between adult and embryonic/larval stages.

#### Growth zone development

**Fig. 14:**
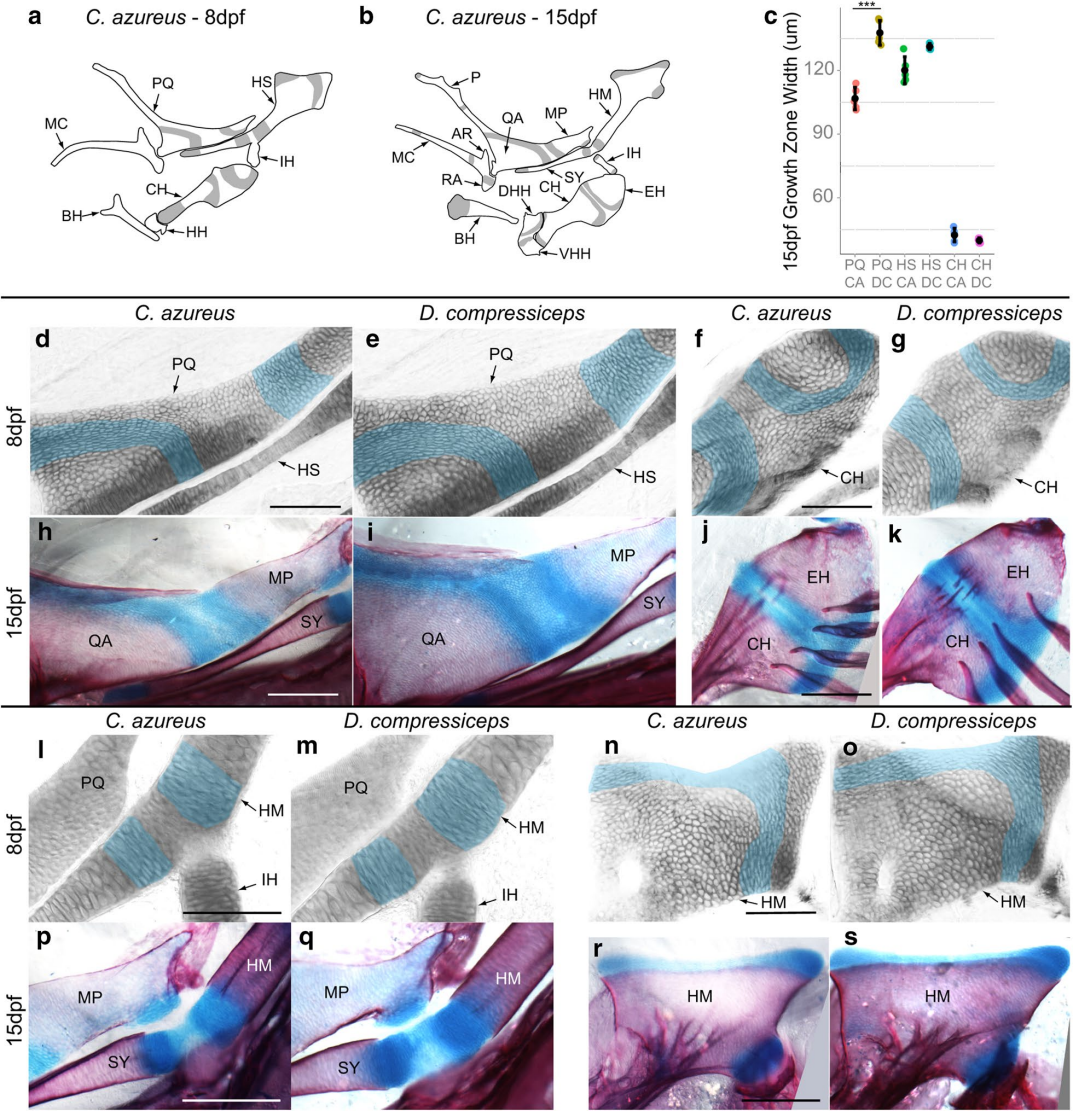
Development of endochondral growth zones in the pharyngeal skeletons of CA and DC larvae. **a**, **b** Camera lucida drawings of first and second pharyngeal arch cartilages in 8 and 15 dpf CA larvae showing endochondral growth zones (*gray*). **a** CA larval cartilages at 8 dpf. **b** Cartilage bones differentiating at 15 dpf from cartilage templates. **c** Mean and distribution of growth zone width in CA and DC in 15 dpf PQ, HS and CH (*n* = 5, each). **d**–**s** Higher magnification images of developing endochondral growth zones in dissected and flat-mounted alizarin red-/alcian blue-stained first and second pharyngeal arch cartilages in 8 dpf (*blue shading* **d**–**g**, **l**–**o**), and 15 dpf (**h**–**k**, **p**–**s**) CA and DC larvae. *Scale bar* 100 um. ***p < 0.001, Tukey’s HSD test. Bone nomenclature after [64]. *AR* articular, *BH* basihyal, *CH* ceratohyal, *DHH* dorsal hypohyal, *EH* epihyal, *HH* hypohyal, *HM* hyomandibular, *HS* hyosymplectic, *IH* interhyal, *MC* Meckel’s, *MP* metapterygoid, *MX* maxilla, *P* palatine, *PQ* palatoquadrate, *QA* quadrate, *RA* retroarticular, *SY* symplectic, *VHH* ventral hypohyal

Because of the central role played by skeletal growth zones in post-embryonic skeletal development, we investigated the timing of their appearance during embryonic stages. The first cellular rearrangements indicative of growth zones were observed at 8 dpf in pharyngeal cartilages and were most obvious in larger, anterior elements: PQ, HS and CH (Fig. 14). By 8 dpf fields of chondrocytes flattened along semicircular patterns within these elements (Fig. 14d–g, l–o). This stereotypical pattern of cartilage development is characteristic of both growth plates in mammalian long bones and craniofacial cartilage development in larval zebrafish [41, 44]. The spatial layout of these arrays corresponded to those of future growth zones (Fig. 14a, b, h–k, p–s). At this stage, two obvious regions of coordinated chondrocyte flattening were observed in PQ, four in HS and three in the CH of both CA and DC (Fig. 14a). Chondrocyte hypertrophy and ossification were later observed (15 dpf) on one side of each of these arrays, as revealed by alizarin red-positive staining (Fig. 14a, b, h–k, p–s). Quantification of growth zone width in PQ, HS and CH revealed significant differences in width between CA and DC: Growth zone width was 29.0 % larger in PQ of DC than of CA and 9.3 % larger in HS of DC than of CA, while it was 5.9 % smaller in CH of DC (Fig. 14c; Tukey’s HSD; Table 2). While the significance of growth zone size remains to be determined, a reasonable interpretation is that wider growth zones may produce faster growth via a larger number of proliferative chondrocytes. In addition, the wider PQ and HS growth zones of DC correlate with the larger size of their derivative adult elements in DC.

## Conclusions

### Phenotypic integration and modular organization in the cichlid skull

**Fig. 15:**
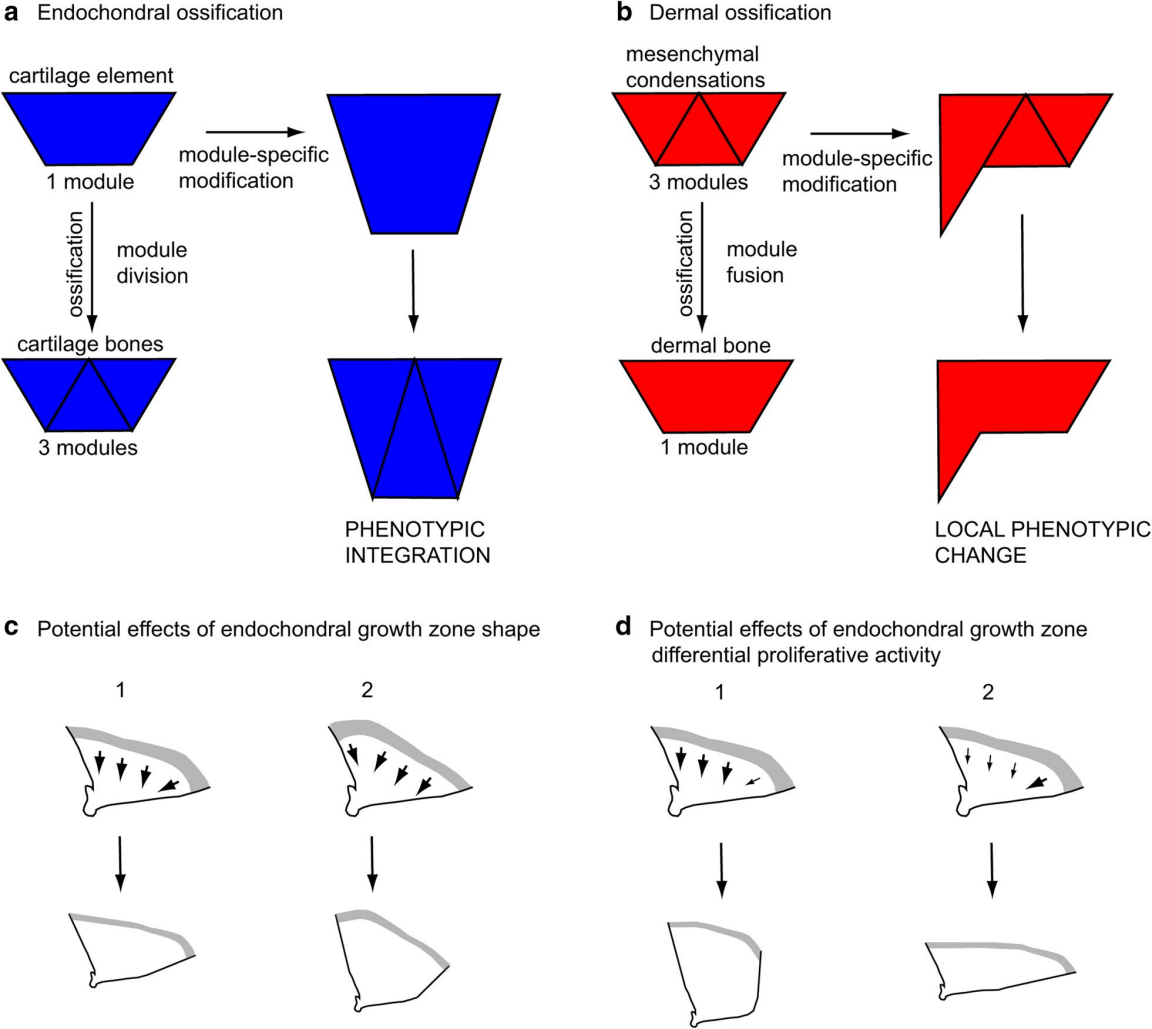
Effects of ossification mode on phenotypic integration and of endochondral growth zone shape and proliferative activity on adult morphology. **a** A simple module-specific change at the cartilage stage of skeletal development results in phenotypically integrated morphological modifications in all three derived cartilage bones. **b** A simple module-specific change at the condensation stage of skeletal development results in a spatially restricted morphological modification within the derived dermal bone. **c** Hypothetical effects of growth zone shape on QA morphology. **d** Hypothetical effects of differential proliferative activities within the QA endochondral growth zone on adult morphology. Growth zone represented as *gray area*. *Arrow direction* indicates growth direction; *arrow thickness* indicates proliferative activity at growth zone. *QA* quadrate

Two cranial ossification mechanisms appear to produce separate patterns of skeletal element integration that underlie morphological differences in the crania of *C. azureus* and *D. compressiceps.* Endochondral ossification tends to produce integrated changes in multiple skeletal elements derived from, or whose development is intimately associated with, single precursor cartilages (Fig. 15a). The VO, ME and LE bones, for example, undergo coordinated shape and positional changes that directly correspond to differences in the development of the ethmoid cartilage. In contrast, dermal bones are prone to region-specific changes within individual skeletal elements (Fig. 15b). The PMX, for example, develops via dermal ossification, and the length of its dentigerous process varies independently of that of its ascending arm.

This opposition in modular development—division of a module into smaller ones (cartilage bone) or fusion of small modules into a larger one (dermal bone), provides distinct properties of phenotypic integration likely to influence the evolvability of skeletal morphology (Fig. 15a, b). In the head, the strategic placement of cartilage versus dermal bones provides distinct opportunities to evolve novel shapes. We speculate that cartilage bones give the opportunity to achieve integrated changes in the morphology of all their derivatives, which may be useful to create changes in overall head shape rapidly, such as length, height and/or width, but constrain possibilities for bone-specific shape evolution (Fig. 15a). In contrast, developmental changes in any of the distinct ossification centers that form individual dermal bones have the potential to produce novel individual bone shapes that may not be integrated with surrounding elements, but which may be useful where subtle changes in individual bone shape influence function, such as in jaw elements (Fig. 15b).

Phenotypic integration derived from the modular organization of complex structures has been a defining aspect of the developmental module concept since its formulation [45], and the mouse lower jaw skeleton is an established model to study the relationships between modularity and phenotypic integration [46–48]. However, the contrasting modular architecture of cartilage and dermal bones highlighted in our study provides a novel perspective on how development may have constrained the rapid diversification of craniofacial morphologies in East African Rift lake cichlids.

### Role of skeletal growth zones in cichlid craniofacial evolution

Our findings also suggest that evolutionary changes in skeletal development at embryonic/larval stages become amplified by subsequent growth that generates profound differences in adult skeletal morphology, such as those we observe between DC and CA. A similar conclusion was recently drawn from the comparative analysis of shape ontogeny in six other Lake Malawi cichlids with distinct head morphologies [29]. We speculate that this amplification occurs at spatially restricted growth zones, the properties of which are dictated by the type of bone they produce: dermal or cartilage bone. Cartilage bone growth occurs at endochondral growth zones, as exemplified for the QA and MP bones, and our results suggest that these zones influence skeletal shape and size through both their shape, which is patterned during embryonic and larval development, and their proliferative activity, (Fig. 15c, d). In addition, element-specific differences in cartilage proliferation may produce overall head shape differences. Cell proliferation assays will help determine how growth zones contribute to skeletal shape and size differences in the cichlid head.

In contrast, dermal bone outgrowth occurs through a variety of mechanisms in which deposition patterns of new mineralized matrix by osteoblasts differ. The best-characterized pattern of tetrapod dermal bone growth occurs at the edges of developing cranial bones. The profound shape changes occurring in the DC skull vault beyond embryonic development suggest an important role for differential growth at cranial sutures. Studies of the zebrafish operculum have revealed a similar pattern, where an increase in dermal bone size by osteoblasts leaves incremental growth bands in the bone matrix, while two other modes, “spur” and “veil” formation, produce new shapes with only minor increases in bone size [49]. Many dermal bones differ in shape or size between DC and CA, the most spectacular being the dentary, and future studies will have to determine the location and mode(s) of bone outgrowth for each element.

### Candidate developmental pathways underlying cichlid craniofacial evolution

The morphologically diverse cichlid species assemblies populating the Rift Lakes of East Africa have been described as natural mutant panels which can offer insight into the identity of genetic variants underlying adaptive phenotypic changes [50]. This “evolutionary mutant model” approach complements the phenotypic analyses of induced mutations in conventional model organisms to explore phenotype to genotype connections [51]. Although some loci without prior association with craniofacial development have been discovered in cichlids [28], polymorphisms mapped in cichlids and other natural populations are often associated with developmental pathways previously described in model organisms: Hedgehog, Wnt, Bmp signaling, etc. [26, 27, 52, 53]. The novelty of these natural variants is that they often reveal functionally important cis-regulatory elements [54, 55] and groups of loci that affect phenotypes in a quantitative manner. Furthermore, the phenotypic changes that they induce are functionally integrated into organisms in a way that does not impair their ability to survive. Ultimately, quantitative genetic studies of craniofacial traits in cichlids will fill a large knowledge gap in the structure and function of the gene regulatory networks (GRN) that control skull morphogenesis.

Our descriptive analysis of major trait differences between CA and DC and the ontogeny of these characters points toward several known developmental pathways, some of which act during embryonic patterning of the pharyngeal arches and palate region. For instance, the specific enlargement of cartilage elements derived from the dorsal first and second pharyngeal arches, the palatoquadrate and hyosymplectic, may result from divergence in the dorso-ventral pharyngeal patterning GRNs. As interactions between Edn1, Bmp and Wnt pathways pattern dorsal pharyngeal domains by restricting the ventral extant of *jag1b* expression [56–58], we speculate that this GRN may underlie expansion of dorsal elements by either ventrally stretching the *jag1b* expression domain, or stimulating increased proliferation of *jag1b*-expressing skeletal progenitors. Similarly, differences in the palate developmental program, which is regulated by Shh and Pdgf signaling, may underlie differences in ethmoid cartilage size between DC and CA and the preorbital region expansion of the cichlid neurocranium [59–61].

The F1 progeny examined here is the product of a mapping cross between CA and DC that we are currently using to identify: (1) the loci underlying craniofacial morphological divergence between these two species and (2) the developmental pathways underlying the adaptive radiations of East African Rift Lake Cichlids. This cross holds promise for contributing to our understanding of cichlid skull evolvability since the cranial differences between these species (Figs. 1, 2) are strongly associated with the most important axis of morphological evolution that has arisen in all Rift Lake cichlid radiations: divergence in preorbital size [22, 24]. We are currently examining the F2 progeny of this cross at multiple developmental stages. In combination with our detailed developmental descriptions of both parental species, this should allow us to gain insight into the genetic controls of bone development in multiple cranial regions and their connection to cichlid skull evolvability.

## Methods

### Animal care

DC and CA brood stocks were purchased from pet stores and maintained separately at 28 °C in 60-gallon tanks. DC offspring were obtained from breeding tanks housing DC adults alone and CA offspring from those containing CA adults alone. Hybridization was achieved by crossing a DC male with a CA female in a 60-gallon tank containing a total of five CA females. Mouth-brooding females were separated from tank mates with a tank partition. Juvenile offspring were collected at 2 weeks post-fertilization and grown in 10-gallon tanks for 2–3 months, after which they were grown for a year in a 60-gallon tank. Juveniles were fed a mixture of live brine shrimp and crushed Hikari Cichlid Gold baby pellets, followed by baby pellets alone, mini pellets and finally medium pellets, depending on specimen age/size. All adults were fed Hikari Cichlid Gold medium pellets.

### Skeletal preparations

Forty-three adult specimens were used in this study: 12 DC individuals, 15 CA individuals and 16 F1 hybrids. Adult specimens of mixed sexes were collected at 12 months of age and older (CA: 5.7 cm < SL < 10.3 cm; DC: 9.3 < SL < 15.4; F1: 6.4 cm < SL < 11.0 cm), killed with tricaine as approved by the UCI IACUC and fixed for 3–7 days in 10 % formalin. Specimen heads were then skinned and skeletal staining was performed as described by [62]. Internal skeletal elements (ceratohyal and gill arch complexes) were dissected in five individuals of each species/line. Bone type and abbreviations are indicated in Table 3.

**Table 3:** Skeletal region, name, type and abbreviation for each bone examined

Region	Bone name	Bone type	Abbreviation
Neurocranium			
Olfactory	Vomer	D	VO
	Mesethmoid	C	ME
	Lateral ethmoid	C	LE
Orbital	Frontal	D	FR
	Parasphenoid	D	PS
	Basisphenoid	C	BS
Otic	Parietal	D	PA
	Sphenotic	C	SO
	Epiotic	C	EO
	Pterotic	C	PO
	Prootic	C	PRO
Occipital	Supraoccipital	D	SOC
	Basioccipital	C	BO
	Exoccipital	C	EOC
Mandibular arch	Premaxilla	D	PMX
	Maxilla	D	MX
	Dentary	D	DE
	Articular	C	AR
	Palatine	C	P
	Ectopterygoid	D	EP
	Entopterygoid	D	ENP
	Quadrate	C	QA
	Metapterygoid	C	MP
Hyoid arch	Hyomandibular	C	HM
	Symplectic	C	SY
	Interhyal	C	IH
	Epihyal	C	EH
	Ceratohyal	C	CH
	Dorsal hypohyal	C	DHH
	Ventral hypohyal	C	VHH
	Basihyal	C	BH
	Operculum	D	OP
	Preoperculum	D	POP
	Suboperculum	D	SOP
	Interoperculum	D	IOP
	Branchiostegal rays	D	BSR
Gill arches	Pharyngobranchial (1–4)	C	PB
	Upper Tooth Plate (2–4)	D	UTP
	Epibranchial (1–4)	C	EB
	Ceratobranchial (1–5)	C	CB
	Lower tooth Plate	D	LTP
	Hypobranchial (1–3)	C	HB
	Basibranchial (1–4)	C	BB

Embryos and larvae were also killed with tricaine as approved by the UCI IACUC, at 5 days post-fertilization (dpf)/1 caudal fin ray elements (CFRE), 8 dpf/3 CFRE and 15 dpf/9 CFRE [42] and fixed in 4 % paraformaldehyde (PFA) for 3 days. The 15dpf/9 CFRE stage was chosen primarily because it precedes yolk depletion by approximately a day, making growth up to this stage independent of food intake. Specimens were cleared and stained as described in [63], and five specimens of each species were analyzed for each stage. Adult skeletal nomenclature follows [40].

### Image capture

Images of whole adult specimens were captured using a Panasonic GH1 camera with a Panasonic Lumix G 20 mm f/1.7 lens. Images of dissected skeletal elements from the neurocrania of adults, larvae and embryos were captured using an Olympus SZX12 dissecting microscope equipped with an Olympus DF PLAPO 1.2 × PF2 objective and an Olympus DP70 camera using DP-Controller software (Olympus). Images of flat-mounted embryos and larvae were captured with a Zeiss Axioplan 2 microscope equipped with Plan-NEOFLUAR objectives, and a Micropublisher 5.0 RTV camera using Volocity software (Improvision). ImageJ was used for all linear and surface area measurements. Landmark positions were also recorded as (*x*, *y*) coordinates in ImageJ.

### Data transformations, geometric morphometrics and statistics

Individual differences in size, orientation and position were first minimized by performing a Procrustes superimposition of the landmarks of all adult specimens using the program CoordGen8. Procrustes transformations superimpose the landmarks of all specimens as much as possible and scale the landmark panels of each individual to the same centroid size. The software CVAGen8 was then used to perform a canonical variance (CV) analysis to identify canonical variate axes that best discriminated the three groups of landmark configurations: CA, DC and F1. Deformation vectors representing the variation identified by a given CV axis around a mean landmark configuration were also visualized in CVAGen8.

Linear measurements between landmarks in adult specimens or on dissected skeletal elements were plotted against individual standard length (SL) and backtransformed to a SL of 10 cm. Linear measurements in embryos and larvae were not transformed.

Data were graphed using ggplot2 (RStudio), and Tukey’s honest significant difference (HSD) tests were performed using TukeyHSD (RStudio).

## Abbreviations

AR: articular
BB: basibranchial
BH: basihyal
BO: basioccipital
BS: basisphenotic
BSR: branchiostegal ray
CA: *Copadichromis azureus*
CB: ceratobranchial
CH: ceratohyal
CV: canonical variate
DC: *Dimidiochromis compressiceps*
DE: dentary
DHH: dorsal hypohyal
EB: epibranchial
EH: epihyal
EOC: exoccipital
EO: epioccipital
ENP: entopterygoid
EP: ectopterygoid
FR: frontal
GRN: gene regulatory network
HB: hypobranchial
HH: head height
HL: head length
HM: hyomandibular
HSD: honest significant difference
HW: head width
IH: interhyal
IOP: interoperculum
LE: lateral ethmoid
LTP: lower tooth plate
ME: mesethmoid
MP: metapterygoid
MX: maxilla
OP: operculum
P: palatine
PA: parietal
PB: pharyngobranchial
PMX: premaxilla
PO: pterotic
POP: preoperculum
PRO: prootic
PS: parasphenoid
QA: quadrate
SO: sphenotic
SOC: supraoccipital
SOP: suboperculum
SL: standard length
SY: symplectic
TL: trunk length
UTP: upper tooth plate
VHH: ventral hypohyal
VO: vomer
Z1–3: zone 1–3
